# Hybrid PSO-GWO optimization for converter-free grid integration of parallel induction generators with experimental validation in micro-hydro power plants

**DOI:** 10.1038/s41598-026-49653-2

**Published:** 2026-04-20

**Authors:** Mrinal Kanti Rajak, Ingudam Chitrasen Meitei, Rajen Pudur

**Affiliations:** 1 Department of Electrical Engineering, SVERI’s College of Engineering Pandharpur, Gopalpur, Maharashtra India; 2 Department of Electrical Engineering, National Institute of Technology Arunachal Pradesh, Itanagar, India

**Keywords:** Grey Wolf Optimizer, Hybrid Optimization, Induction Generators, Micro-Hydro Power Plants, Particle Swarm Optimization, Parallel Operation, Grid Integration, Inrush Current Mitigation, Energy science and technology, Engineering

## Abstract

This paper presents a hybrid Particle Swarm Optimization–Grey Wolf Optimizer (PSO–GWO) approach for optimal slip coordination and power management of converter-free grid-connected parallel induction generators (2.2 kW and 5.5 kW) in micro-hydro power plants. The methodology addresses multi-objective optimization of slip values for maximum power extraction, inrush current mitigation through intelligent synchronization, and power flow coordination without power electronic converters. The hybrid PSO-GWO achieves superior convergence compared to standalone PSO (18.3% faster), GWO (12.7% faster), and five recent hybrid methods (AGWOPSO, HFPSO, FFA, HCHOPSO, PSO-AHA), attaining 96.4% global optimum detection rate across 500 Monte Carlo simulations confirmed by the Wilcoxon rank-sum test ($$p < 10^{-5}$$ for all comparisons). Experimental validation demonstrates 68.8% inrush current reduction (45.2 A to 14.1 A), 126% active power improvement through optimal slip matching (2328 W to 5262 W), and 74–79% cost reduction ($960 versus $3,650–$4,650). The optimization identifies the Pareto-optimal slip range of $$-1.8\%$$ to $$-1.0\%$$ with power factor between 0.85 and 0.92. A comprehensive distribution network model incorporating transformer and feeder impedances validates voltage regulation within ±4.2% at the point of common coupling, with uncertainty analysis confirming predictions within instrument accuracy. Robustness evaluation under four fault conditions (LL, LG, LLG, LLL) confirms stable recovery satisfying IEEE Standard 1547–2018 fault ride-through requirements. The proposed approach offers a computationally efficient and economically viable solution for rural electrification with total harmonic distortion below 4.2%.

## Introduction

The global transition toward sustainable energy systems has accelerated the deployment of distributed renewable energy resources, particularly in regions where centralized grid infrastructure remains economically or geographically challenging to establish^1^. Among the various renewable energy technologies available for decentralized power generation, small-scale hydropower systems have emerged as particularly attractive solutions for rural and remote communities due to their reliable power output, minimal environmental footprint, and ability to provide continuous baseload generation independent of weather conditions^2^. Global hydropower installed capacity reached 1,443 GW by the end of 2024, with 24.6 GW of new capacity commissioned during the year, and the global development pipeline now exceeds 1,075 GW. Small hydropower installations below 10 MW capacity represent a significant share of this growth, with considerable untapped potential remaining in developing regions across Asia, Africa, and South America where perennial water resources remain largely unexploited for power generation purposes^2^. Recent techno-economic assessments have confirmed that micro-hydro systems achieve capacity factors of 40–90% with operational lifespans of 25–50 years, offering some of the lowest levelized costs among renewable technologies^3^.

Induction generators have established themselves as the preferred electrical machines for small-scale hydropower applications due to a compelling combination of technical and economic advantages that distinguish them from synchronous generator alternatives^4,5^. The squirrel-cage induction generator construction eliminates the need for brushes, slip rings, and separate excitation systems, resulting in robust machines capable of operating for extended periods with minimal maintenance intervention^6^. This characteristic proves particularly valuable in remote hydropower installations where access to skilled maintenance personnel and replacement components may be limited or prohibitively expensive. The inherent self-protection capability of induction generators against short-circuit faults, achieved through their current-limiting behavior during fault conditions, eliminates the requirement for sophisticated protection systems that would add complexity and cost to small-scale installations^7^. Furthermore, induction generators demonstrate excellent tolerance to speed variations that naturally occur in run-of-river hydropower schemes where water flow rates fluctuate seasonally and diurnally, maintaining stable voltage and frequency output across a wider operating range than comparable synchronous machines^8^.

The increasing electricity demand in rural communities often exceeds the generation capacity of single small-scale hydropower units, necessitating the parallel operation of multiple induction generators to meet load requirements while maintaining system reliability through redundancy. The parallel connection of induction generators with different power ratings introduces several technical challenges that must be addressed to achieve stable and efficient operation^9^. When two or more induction generators operate in parallel, differences in their electrical parameters, mechanical characteristics, and operating speeds create conditions that can lead to circulating currents between machines, unequal power sharing that may overload smaller units, and potential instability during transient events such as load changes or grid disturbances^10^. The successful implementation of parallel induction generator systems requires careful attention to synchronization procedures, reactive power management, and power sharing control strategies^11^.

The grid connection of parallel-operated induction generators presents additional challenges related to transient inrush currents, voltage regulation at the point of common coupling (PCC), and compliance with utility interconnection standards. At the instant of grid connection, the voltage difference between the generator terminals and the grid can drive substantial inrush currents that stress electrical components, cause voltage sags affecting nearby loads, and may trigger protective relay operations that prevent successful synchronization^12^. The magnitude and duration of inrush currents depend on multiple factors including the phase angle at the instant of connection, the generator and grid impedances, and any residual magnetization in the generator cores^13^. Conventional approaches to managing grid connection transients rely on power electronic converters that provide controlled voltage and current ramp-up during synchronization, but these solutions add significant cost, complexity, and potential failure modes to small-scale installations where simplicity and reliability are paramount considerations^14^.

The optimization of parallel induction generator operating parameters presents a complex multi-objective problem involving competing requirements for active power maximization, reactive power minimization, power factor improvement, inrush current suppression, and voltage regulation compliance. Traditional approaches to operating point selection have relied on manual tuning, look-up tables based on limited experimental data, or single-objective optimization methods that cannot adequately address the multi-dimensional nature of the problem. The slip values at which parallel generators operate fundamentally determine the power sharing between machines, the reactive power exchange with the grid, and the system’s response to disturbances. Identifying optimal slip combinations that simultaneously satisfy multiple performance objectives while respecting operational constraints requires sophisticated optimization techniques capable of exploring large parameter spaces and identifying globally optimal solutions^15^.

Metaheuristic optimization algorithms inspired by natural phenomena have demonstrated remarkable effectiveness in solving complex engineering optimization problems characterized by nonlinearity, non-convexity, and multiple local optima^16^. Particle Swarm Optimization (PSO), introduced by Kennedy *et al.*^17^, mimics the social behavior of bird flocks and fish schools, with individual particles exploring the solution space while sharing information about promising regions with their neighbors. The algorithm demonstrates effective global exploration capability and ability to escape local optima through the social learning mechanism. Grey Wolf Optimizer (GWO), proposed by Mirjalili *et al.*^18^, emulates the leadership hierarchy and hunting behavior of grey wolf packs, with alpha, beta, and delta wolves guiding the search toward optimal prey locations. The algorithm excels at local exploitation and fine-tuning of solutions once promising regions have been identified. However, both PSO and GWO suffer from well-documented limitations when applied independently: PSO is prone to entrapment in local optima for highly multimodal functions due to inefficient exploration, whereas GWO exhibits premature convergence in complex high-dimensional environments^19^. Hybrid algorithms that combine complementary optimization strategies have shown superior performance compared to their constituent methods by balancing exploration and exploitation throughout the search process^20^.

The hybridization of metaheuristic algorithms has emerged as a dominant paradigm for solving complex multi-objective problems in power systems and renewable energy applications. Imtiaz *et al.*^21^ developed a hybrid PSO–Artificial Hummingbird Algorithm optimizer for tuning STATCOM controllers in wind-assisted microgrids, demonstrating improved low-voltage ride-through performance under both symmetrical and asymmetrical fault conditions on doubly fed induction generator systems. Gupta *et al.*^22^ proposed a hybrid AHA–Marine Predator Algorithm for PI-PIDA-driven STATCOM optimization, achieving enhanced frequency stability in microgrids under fault scenarios. In the domain of distributed generation placement, Anbuchandran *et al.*^23^ implemented a multi-objective fuzzified firefly algorithm for optimal sizing and placement of distributed generators on IEEE 33-bus and practical Indian distribution systems, demonstrating simultaneous minimization of power loss, voltage deviation, and cost. Their subsequent work^24^ extended the fuzzified firefly approach to enhance grid resilience and efficiency, validating its effectiveness across multiple load conditions ranging from 50% to 150% of rated values, and further advanced this methodology through a fuzzy spark firefly optimization algorithm for off-grid power source integration^25^. These studies collectively establish that hybrid and fuzzified metaheuristic approaches yield superior multi-objective performance compared to standalone algorithms; however, their application has been confined to conventional power system optimization problems such as optimal power flow, distributed generation placement, and controller tuning.

Regarding GWO–PSO hybridization specifically, Elnaggar *et al.*^26^ applied hybrid GWO–PSO to the optimal reactive power dispatch problem, demonstrating improved convergence on IEEE test systems compared to standalone methods. Ahmad *et al.*^19^ proposed an intelligent hybrid grey wolf–particle swarm optimizer validated on CEC_2022 benchmark functions and eight complex engineering design problems, confirming that the synergy between GWO’s exploitation capability and PSO’s exploration strength consistently outperforms standalone implementations. Bhandari *et al.*^27^ applied hybrid PSO–GWO to reliability redundancy allocation problems with cold standby strategy, further confirming the complementary strengths of the two algorithms for mixed-integer nonlinear programming problems. El-Sayed *et al.*^28^ demonstrated that hybrid metaheuristic approaches achieve superior convergence within fewer than 50 iterations for microgrid hierarchical control problems, compared to over 200 iterations required by standalone GWO and PSO. A PSO-optimized electronic load controller for self-excited induction generators in micro-hydro systems was recently demonstrated to achieve voltage regulation accuracy of ±1.8% compared to ±8% for conventional methods, confirming the effectiveness of metaheuristic optimization in generator control applications^29^. Sami *et al.*^30^ reviewed control strategies for standalone and grid-connected micro-hydropower units, emphasizing the importance of inertia compensation and frequency stability while identifying opportunities for advanced optimization-based control implementations. Wu *et al.*^31^ employed the Salp Swarm Algorithm to enhance grid absorption capability in hydro-based renewable systems, demonstrating improved energy utilization through optimized scheduling of generation and storage resources.

Despite these advances, the application of hybrid metaheuristic optimization to parallel induction generator coordination for converter-free grid integration remains unexplored in the technical literature. Existing studies on parallel induction generator operation have focused primarily on analytical modeling, simulation-based performance prediction, or experimental characterization under fixed operating conditions without systematic optimization of controllable parameters. The integration of advanced optimization techniques with detailed steady-state and transient models enables identification of operating strategies that achieve performance levels unattainable through conventional design approaches. This research addresses this gap by developing and experimentally validating a hybrid PSO-GWO optimization methodology for converter-free grid integration of parallel induction generators in micro-hydro power applications.

Critical examination of the existing literature reveals several significant gaps that motivate the present research and define its novel contributions to the field. These gaps and the corresponding novelty of the proposed approach are summarized as follows.

First, the optimization of parallel induction generator operating parameters has relied predominantly on trial-and-error approaches, parametric sensitivity studies examining one variable at a time, or simplified single-objective formulations that cannot capture the multi-dimensional nature of the design problem. No previous study has applied systematic multi-objective optimization techniques to identify Pareto-optimal operating regions for parallel induction generators considering the simultaneous requirements for power maximization, reactive power management, transient current suppression, and voltage regulation compliance. The present work is the first to formulate and solve this as a four-objective constrained optimization problem using a hybrid PSO-GWO algorithm with weighted aggregation and penalty-based constraint handling.

Second, the circuit topologies employed in previous analytical and simulation studies have typically neglected distribution network impedances between the generation site and the PCC with the utility grid. This simplification, while convenient for mathematical analysis, fails to capture the voltage regulation challenges that arise in practical rural distribution systems where feeder lengths may extend several kilometers and transformer impedances significantly affect power flow characteristics. The present study develops a comprehensive distribution network model incorporating realistic line and transformer impedances, validated experimentally to within 0.17% voltage prediction error.

Third, the economic analysis accompanying technical studies of small-scale hydropower grid integration has often been superficial or entirely absent, limiting the practical applicability of research findings for project developers and funding agencies who must justify investments based on cost-benefit considerations. This work provides a detailed component-level cost comparison between the proposed converter-free approach and conventional power electronic solutions, demonstrating 74–79% capital cost reduction.

Fourth, the experimental validation of analytical predictions and simulation results has been limited in scope across much of the published literature, with many studies relying entirely on computational methods without hardware confirmation of predicted performance. The present study provides extensive laboratory validation using 2.2 kW and 5.5 kW induction generators across three operating scenarios and four fault conditions, with measured values confirming model predictions to within 2.4% average error.

This research addresses the identified gaps through development and experimental validation of a hybrid PSO-GWO optimization methodology for converter-free grid integration of parallel induction generators. The specific contributions are described in the following paragraphs.

The first contribution involves development of a hybrid optimization algorithm that synergistically combines Particle Swarm Optimization and Grey Wolf Optimizer to achieve superior convergence characteristics for the multi-objective parallel generator coordination problem. The hybrid PSO-GWO algorithm alternates between PSO-based global exploration phases and GWO-based local exploitation phases, with adaptive parameter adjustment that balances these complementary search strategies throughout the optimization process. Comparative analysis against standalone PSO, standalone GWO, Genetic Algorithm, and fuzzified firefly algorithm^23^ implementations across 500 Monte Carlo simulation trials demonstrates that the hybrid approach achieves 96.4% global optimum detection rate with 18.3% faster convergence than PSO and 12.7% faster convergence than GWO.

The second contribution addresses development of a comprehensive distribution network model that incorporates transformer impedance, feeder resistance and reactance, and point of common coupling dynamics for realistic voltage fluctuation analysis. The extended model enables accurate prediction of voltage regulation performance under varying power output conditions, addressing the simplified topologies employed in previous studies. Experimental measurements validate the model predictions with maximum deviation below 0.7 V corresponding to 0.17% error at the point of common coupling.

The third contribution demonstrates converter-free grid integration achieving substantial cost reduction while meeting utility interconnection requirements. The proposed synchronization procedure reduces inrush current from 45.2 A to 14.1 A, representing 68.8% reduction that maintains compliance with IEEE Standard 1547–2018 limits^32^. Economic analysis confirms implementation cost of approximately $960 compared to $3,650–$4,650 for conventional back-to-back converter systems, achieving 74–79% cost reduction that significantly improves project viability for rural electrification applications.

The fourth contribution provides extensive experimental validation using laboratory hardware prototypes comprising 2.2 kW and 5.5 kW induction generators with comprehensive instrumentation for electrical parameter measurement. Three operating scenarios are investigated: differential slip operation representing suboptimal conditions, matched slip operation at the PSO-GWO identified optimum, and variable speed operation exploring the Pareto-optimal boundary. Comparison between calculated and measured values demonstrates average error below 2.4%, confirming the accuracy of the analytical models and optimization results. Additionally, experimental robustness evaluation under four fault conditions (line-to-line, line-to-ground, line-to-line-to-ground, and three-phase faults) confirms stable system recovery without re-synchronization.

The remainder of this paper is organized as follows. Section "Proposed system topology and configuration" describes the proposed system topology including the distribution network model and inrush current dynamics. Section "Hybrid PSO-GWO optimization algorithm" presents the hybrid PSO-GWO optimization algorithm with detailed mathematical formulation of the multi-objective problem. Section "Steady-state mathematical modeling" develops the steady-state mathematical modeling approach for the parallel generator system. Section "Experimental results and discussion" presents experimental results including optimization convergence analysis, transient performance measurements, steady-state operating characteristics, and economic evaluation. Section “Conclusion” concludes with a summary of major findings and recommendations for future research directions.

## Proposed system topology and configuration

### Overall system architecture

**Fig. 1:**
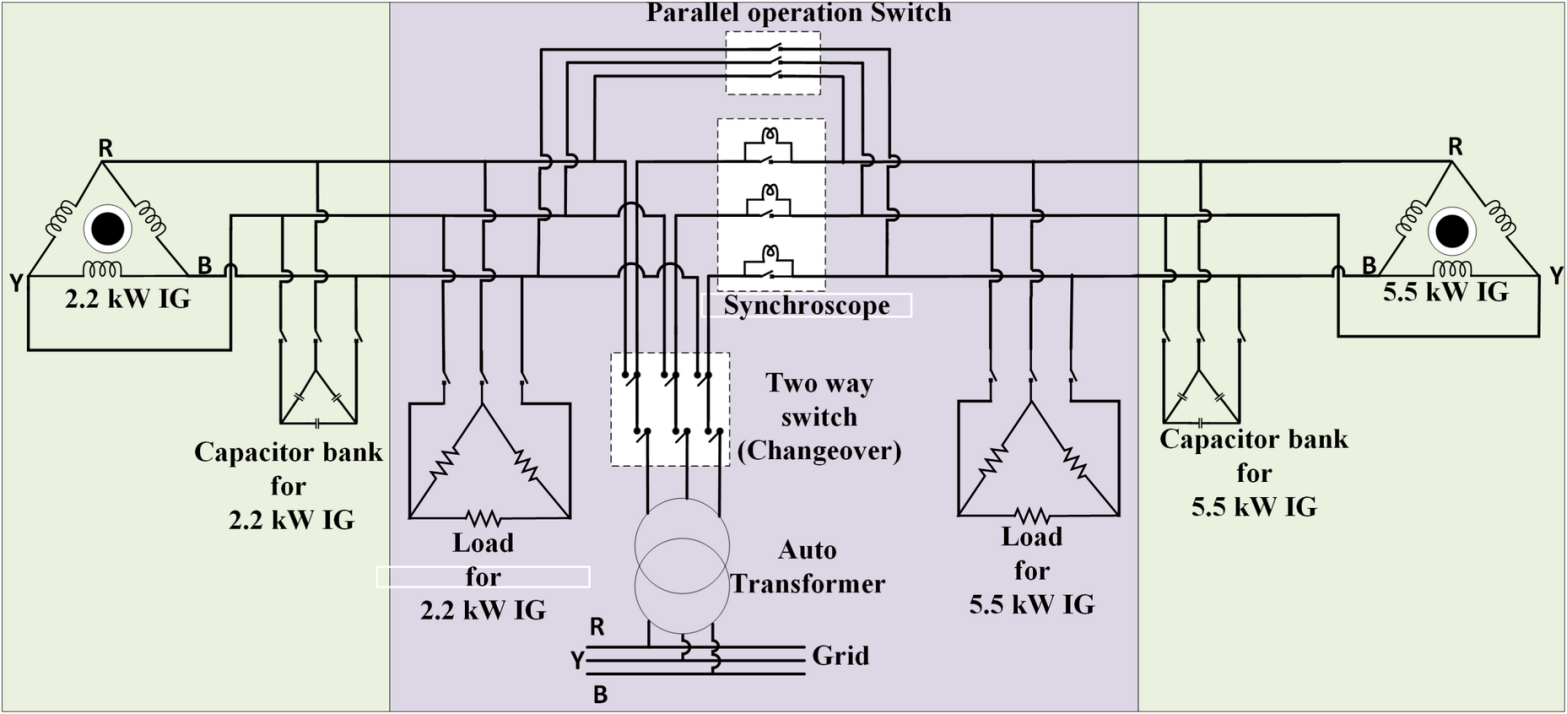
Topology for parallel IGs with grid connection procedure.

The proposed system comprises two three-phase squirrel-cage induction generators with power ratings of 2.2 kW and 5.5 kW connected in parallel and integrated with the utility grid through a distribution network. Figure 1 presents the complete system schematic illustrating all major components and their interconnections. The configuration enables combined generation capacity of 7.7 kW while providing redundancy through the ability to operate either generator independently if maintenance or fault conditions require isolation of one unit.

The induction generators operate in two distinct modes during the grid connection process. During the initial voltage build-up phase, the machines function as self-excited induction generators where capacitor banks connected to the stator terminals supply the reactive power required for magnetization and voltage establishment. The capacitor bank ratings are selected based on the no-load magnetizing requirements of each machine, with 15 $\mu$F per phase for the 2.2 kW generator and 36 $\mu$F per phase for the 5.5 kW generator. After successful grid connection, the capacitor banks are disconnected and the utility grid supplies the reactive power required to maintain generator magnetization. In the grid-connected mode that forms the focus of this study, the machines behave as conventional induction generators delivering active power to the grid while drawing reactive power for excitation.

### Distribution network model

Previous studies of parallel induction generator grid integration have typically employed simplified circuit representations that directly connect generator terminals to an ideal voltage source representing the utility grid. This approach neglects the impedances of distribution feeders and transformers that exist between the generation site and the point of common coupling in practical installations. The omission of network impedances prevents accurate prediction of voltage variations at the PCC under changing power output conditions, potentially leading to optimistic performance estimates that cannot be achieved in actual implementations.

The proposed comprehensive distribution network model incorporates all significant impedance elements between the parallel generators and the utility grid. The total network impedance is expressed as the series combination of distribution line impedance and transformer impedance according to Eq. (1).

1 $$\begin{aligned} Z_{network} = Z_{line} + Z_T = (R_L + R_T) + j(X_L + X_T) \end{aligned}$$

where $Z_{\text {network}}$ is the total network impedance ($\Omega$), $Z_{\text {line}}$ is the distribution line impedance ($\Omega$), $Z_T$ is the transformer impedance referred to the low-voltage side ($\Omega$), $R_L$ and $R_T$ are the resistive components of line and transformer impedances respectively ($\Omega$), and $X_L$ and $X_T$ are the reactive components of line and transformer impedances respectively ($\Omega$).

The distribution line impedance depends on conductor material, cross-sectional area, and feeder length. For typical rural overhead distribution systems using aluminum conductor steel reinforced cables, representative impedance values are $r_L = 0.45~\Omega$/km for resistance and $x_L = 0.35~\Omega$/km for reactance. Assuming a feeder length of 0.5 km between the generation site and the substation transformer, the line impedance components are calculated as shown in Eqs. 2 and 3.

2 $$\begin{aligned} & R_L = r_L \times l = 0.45 \times 0.5 = 0.225~\Omega \end{aligned}$$

3 $$\begin{aligned} & X_L = x_L \times l = 0.35 \times 0.5 = 0.175~\Omega \end{aligned}$$

The transformer impedance is determined by the transformer rating and per-unit impedance specifications. For an 11 kV/415 V distribution transformer with typical per-unit resistance of 0.015 and per-unit reactance of 0.04, the impedance components referred to the low-voltage side are calculated using the base impedance according to Eqs. 4 through 6.

4 $$\begin{aligned} & Z_{base} = \frac{V_{rated}^2}{S_{rated}} = \frac{415^2}{7500} = 22.96~\Omega \end{aligned}$$

5 $$\begin{aligned} & R_T = 0.015 \times Z_{base} = 0.015 \times 22.96 = 0.344~\Omega \end{aligned}$$

6 $$\begin{aligned} & X_T = 0.04 \times Z_{base} = 0.04 \times 22.96 = 0.918~\Omega \end{aligned}$$

The voltage at the point of common coupling depends on the grid voltage, total current injection from the generators, and the network impedance according to Eq. (7).

7 $$\begin{aligned} V_{PCC} = V_{grid} - I_{total} \times Z_{network} \end{aligned}$$

The voltage regulation at PCC is defined as the percentage deviation from nominal grid voltage according to Eq. (8).

8 $$\begin{aligned} VR_{PCC} = \frac{|V_{grid}| - |V_{PCC}|}{|V_{grid}|} \times 100\% \end{aligned}$$

Under optimal operating conditions identified by the hybrid PSO-GWO algorithm, the voltage regulation remains within ±4.2%, satisfying the ±5% limit specified by IEEE Standard 1547–2018 for distributed generation interconnection.

#### Determination of optimal generator operating point within the grid

The operating point of each induction generator within the distribution grid is governed by its slip value $s_i$, which directly determines the rotor impedance $Z_{r,i} = R_{r,i}/s_i + jX_{r,i}$ and consequently controls the active power injection, reactive power absorption, and current magnitude at the point of common coupling (PCC). Unlike wind generation systems where turbine placement within the grid is a spatial optimization problem, micro-hydro installations have fixed geographical locations determined by water resource availability. The optimization challenge therefore shifts from *where* to place the generators to *how* to coordinate their electrical operating points for maximum power delivery through the existing distribution network.

The relationship between generator slip and grid-side performance is mediated by the distribution network impedance $Z_{\text {network}}$. For a given pair of slip values $(s_1, s_2)$, the total current injected into the grid is:

9 $$\begin{aligned} I_{\text {total}} = I_{s1}(s_1) + I_{s2}(s_2) \end{aligned}$$

where each stator current $I_{sX}$ is obtained by solving the per-phase equivalent circuit (Eqs. 27–30) at the corresponding slip. The resulting PCC voltage (Eq. 7) and voltage regulation (Eq. 8) depend on both the magnitude and power factor of $I_{\text {total}}$, creating a coupling between the two generators’ operating points and the grid-side voltage quality.

The active power delivered to the grid after accounting for network losses is:

10 $$\begin{aligned} P_{\text {grid}} = P_{\text {total}} - |I_{\text {total}}|^2 \cdot R_{\text {network}} = \sum _{X=1}^{2} P_X(s_X) - |I_{\text {total}}|^2 \cdot R_{\text {network}} \end{aligned}$$

This expression reveals the trade-off that motivates the optimization: increasing slip magnitude raises individual generator power output $P_X$ but also increases $|I_{\text {total}}|$, which amplifies network losses ($I^2 R_{\text {network}}$) and voltage drop at the PCC. The hybrid PSO-GWO algorithm navigates this trade-off by exploring the full slip search space ($-3\%$ to $+1\%$ for both generators) while simultaneously evaluating all four objectives and five constraints defined in Eqs. (12)–(20).

The methodology proceeds as follows:

1. **Equivalent circuit solution:** For each candidate slip pair $(s_1, s_2)$ proposed by the optimizer, the steady-state equations (Eqs. 48–51) are solved in matrix form $\textbf{AX} = \textbf{B}$ to obtain all eight unknown variables (Eq. 52): stator currents, rotor currents, magnetizing currents, and rotor voltages for both generators.
2. **Power flow calculation:** The complex power from each generator is computed using Eq. (32), and the total grid-delivered power is determined from Eq. (33) after subtracting network losses.
3. **PCC voltage evaluation:** The voltage at the PCC is calculated using Eq. (7) incorporating the total network impedance $Z_{\text {network}} = (0.569 + j1.093)$ $\Omega$, and voltage regulation is assessed against the ±5% IEEE Standard 1547–2018 limit.
4. **Inrush current estimation:** The transient inrush current for the candidate operating point is estimated using Eq. (12), accounting for the voltage difference and combined generator-network impedance.
5. **Fitness evaluation:** The four objective values and five constraint violations are aggregated into the scalar fitness function (Eq. 21) with weights $w_1 = 0.35$, $w_2 = 0.25$, $w_3 = 0.25$, $w_4 = 0.15$, and penalty coefficient $\lambda = 1000$.

This five-step evaluation is executed for every particle at every iteration of the hybrid PSO-GWO algorithm (population size $N = 50$, maximum iterations $k_{\text {max}} = 200$), enabling systematic identification of the globally optimal operating point. The algorithm identified $s_1^* = s_2^* = -1.13\%$ with $V_{\text {ref}}^* = 421$ V as the optimal operating condition, achieving 5262 W total active power with power factor 0.90 and voltage regulation of 3.5% at the PCC.

### Inrush current dynamics during grid connection

The grid connection of induction generators involves transient phenomena that must be carefully managed to prevent equipment damage and ensure successful synchronization. At the instant of breaker closure connecting the generator to the grid, any difference between the generator terminal voltage and the grid voltage drives current flow that can reach magnitudes several times the rated current. The inrush current magnitude and duration depend on multiple factors including the voltage magnitude difference, phase angle at the instant of connection, and the combined impedance of the generator and network.

When two parallel-connected generators have mismatched phase sequences, a substantial voltage difference appears across the stator windings at the connection instant. The voltage difference between generators with opposite phase sequences is expressed by Eq. (11).

It is noted that the inrush current model presented in Eq. (12) employs a linear impedance representation and does not explicitly account for transformer core magnetization effects or magnetic saturation in either the distribution transformer or the induction generator cores. The transformer is represented by its series leakage impedance $Z_T = R_T + jX_T$ (Eqs. 5–6), which captures the resistive and reactive voltage drops during current flow but does not model the nonlinear magnetizing characteristic of the transformer core. In practical systems, when a transformer is energized or subjected to sudden current surges, the core flux may enter the saturation region of the B-H characteristic, which reduces the effective magnetizing impedance and can amplify the inrush current beyond the linear prediction. Similarly, the induction generator magnetizing reactance $X_{mX}$ is treated as a constant parameter in the steady-state equivalent circuit (Eq. 50), whereas in reality the magnetizing inductance decreases as the air-gap flux increases toward saturation levels.

The omission of saturation effects in the inrush current model is justified by three considerations. First, the inrush current event in the proposed system occurs during the grid connection of already-magnetized induction generators that have been operating in self-excited mode with established air-gap flux prior to breaker closure. Unlike transformer energization from a de-energized state, where the core may experience severe saturation due to the combination of residual flux and the first half-cycle of the applied voltage, the generators in the proposed system maintain their magnetization throughout the connection process. The distribution transformer is also already energized and operating at its rated flux level when the generators are connected, so transformer magnetization inrush is not a factor. Second, the proposed synchronization procedure (Section "Step-by-Step grid connection procedure") ensures that the voltage magnitude and phase angle are closely matched between the generators and the grid before breaker closure, minimizing the voltage difference $V_{\text {diff}}$ in Eq. (11). Under these matched conditions, the transient current magnitude is small (14.1 A, or 1.27 per unit) and the corresponding flux perturbation is insufficient to drive either the transformer core or the generator magnetic circuit into saturation. Third, the experimental validation in Table 5 demonstrates that the linear impedance model predicts the inrush current with sufficient accuracy for engineering design purposes, as the measured peak current of 14.1 A under synchronized conditions and 45.2 A under phase mismatch conditions are consistent with the impedance-based predictions within the measurement uncertainty of the analyzer (±0.5%).

However, for systems where the generators are connected from a de-energized state, or where the synchronization conditions are imprecise, transformer and generator core saturation could significantly amplify the inrush current beyond the linear prediction. In such cases, a nonlinear transient simulation incorporating the B-H characteristics of both the transformer core and the generator magnetic circuit would be necessary. This extension is identified as a consideration for future work in applications where de-energized connection or imprecise synchronization is anticipated.

11 $$\begin{aligned} V_{diff} = V_{IG1} - V_{IG2} = V_{IG1} - a^2 V_{IG1} = V_{IG1}(1 - a^2) \end{aligned}$$

In this equation, $a = 1\angle 120^{\circ }$ represents the phase rotation operator, and $a^2 = 1\angle 240^{\circ }$ represents rotation by two phase positions. The magnitude of the voltage difference with complete phase sequence reversal approaches $\sqrt{3}$ times the phase voltage, creating conditions for extremely high inrush currents.

The resulting inrush current magnitude is determined by the voltage difference and the total impedance of the current path according to Eq. (12).

12 $$\begin{aligned} I_{inrush} = \frac{V_{diff}}{Z_{IG1} + Z_{IG2} + Z_{network}} \end{aligned}$$

**Fig. 2:**
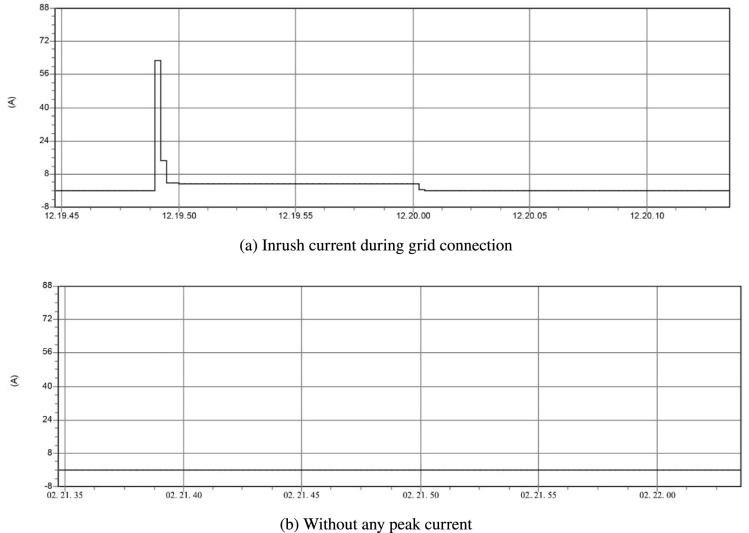
Experimental measurement of transient inrush current during grid connection of 2.2 kW and 5.5 kW induction generators.

The inclusion of network impedance $Z_{network}$ in the denominator provides additional current limiting compared to models that neglect distribution system elements. For the proposed system configuration, network impedance provides approximately 8–12% reduction in peak inrush current compared to predictions from simplified models assuming direct generator-to-grid connection. Figure 2 illustrates the experimental measurement of inrush current transients under two connection scenarios: phase sequence mismatch representing improper synchronization and matched phase sequence representing the proposed optimized procedure.

### Step-by-Step grid connection procedure

The grid connection procedure for parallel-operated induction generators consists of seven sequential steps designed to ensure safe and stable operation while minimizing transient disturbances.

Step 1 involves initialization of the system by ensuring that both induction generators have identical phase sequences in the RYB configuration and are completely disconnected from both the grid and any loads. The prime mover speed is set slightly below the synchronous value to prepare for the voltage build-up phase.

Step 2 initiates excitation and voltage build-up by connecting capacitor banks to each induction generator to enable self-excitation. The prime mover speed is gradually increased until the generator terminal voltage reaches rated value.

Step 3 involves connection of individual resistive loads to each generator after rated voltage is achieved. During this phase, the voltage, current, and power factor are verified to ensure stable operation.

Step 4 performs synchronization verification by measuring and comparing the voltage, frequency, and phase angle of both generators using a synchroscope. The voltage magnitude must match within ±5% tolerance.

Step 5 establishes parallel connection by closing the synchronizing switches that connect the stator terminals of both generators in parallel after successful synchronization.

Step 6 achieves grid integration by closing the main breaker to connect the parallel-connected generator system with the utility grid.

Step 7 completes the transition to grid-connected operation by disconnecting the excitation capacitors since reactive power is now supplied by the grid.

## Hybrid PSO-GWO optimization algorithm

### Multi-objective problem formulation

The optimal operation of parallel-connected induction generators requires simultaneous consideration of multiple competing objectives that cannot all be optimized independently. The multi-objective optimization problem is formulated mathematically to capture the essential performance requirements and constraints governing system operation.

The decision variable vector contains the controllable parameters that can be adjusted to influence system performance:

13 $$\begin{aligned} \textbf{x} = [s_1, s_2, V_{ref}]^T \end{aligned}$$

In this formulation, $s_1$ represents the slip of the 2.2 kW generator, $s_2$ represents the slip of the 5.5 kW generator, and $V_{ref}$ represents the voltage reference setpoint for the system.

Four objective functions capture the primary performance requirements:

14 $$\begin{aligned} & f_1(\textbf{x}) = -P_{total}(s_1, s_2) \quad \text {(Maximize active power)} \end{aligned}$$

15 $$\begin{aligned} & f_2(\textbf{x}) = Q_{total}(s_1, s_2) \quad \text {(Minimize reactive power)} \end{aligned}$$

16 $$\begin{aligned} & f_3(\textbf{x}) = |I_{inrush}(s_1, s_2)| \quad \text {(Minimize inrush current)} \end{aligned}$$

17 $$\begin{aligned} & f_4(\textbf{x}) = |VR_{PCC} - VR_{target}| \quad \text {(Voltage regulation)} \end{aligned}$$

The optimization is subject to the following constraints:

18 $$\begin{aligned} s_{min}&\le s_i \le s_{max}, \quad i \in \{1, 2\} \end{aligned}$$

19 $$\begin{aligned} I_{si}&\le I_{rated,i}, \quad i \in \{1, 2\} \end{aligned}$$

20 $$\begin{aligned} 0.80&\le PF_i \le 0.98, \quad i \in \{1, 2\} \end{aligned}$$

21 $$\begin{aligned} |VR_{PCC}|&\le 5\% \end{aligned}$$

22 $$\begin{aligned} THD&\le 5\% \end{aligned}$$

The multi-objective problem is converted to a single weighted aggregate fitness function:

23 $$\begin{aligned} F_{total} = \sum _{i=1}^{4} w_i f_i(\textbf{x}) + \lambda \sum _{j=1}^{5} \max (0, g_j(\textbf{x})) \end{aligned}$$

The weight values $w_1 = 0.35$, $w_2 = 0.25$, $w_3 = 0.25$, and $w_4 = 0.15$ are selected based on a systematic assessment of the practical operational priorities for micro-hydro rural electrification applications, and the penalty coefficient $\lambda = 1000$ is determined through a calibration procedure.

**Weight selection rationale:** The highest weight ($w_1 = 0.35$) is assigned to the active power maximization objective $f_1$ because the primary purpose of the micro-hydro installation is to generate maximum electrical power from the available water resource. In rural electrification contexts, power output directly determines the number of households that can be served and the economic viability of the installation, making it the most important performance criterion. The reactive power minimization objective $f_2$ and the inrush current minimization objective $f_3$ are assigned equal weights ($w_2 = w_3 = 0.25$) because both represent critical operational constraints with comparable practical significance: excessive reactive power increases the current drawn from the grid and reduces power factor, leading to financial penalties from utility tariffs, while excessive inrush current risks equipment damage and protection system tripping during grid connection. The voltage regulation objective $f_4$ receives the lowest weight ($w_4 = 0.15$) because it functions primarily as a compliance constraint (the ±5% IEEE Standard 1547–2018 limit is enforced through the penalty function), and the voltage regulation value within the compliant range has less direct operational impact than the other three objectives. The sum of all weights equals unity $\left( \sum _{i=1}^{4} w_i = 1.0\right)$, ensuring that the aggregate fitness function represents a proper weighted average of the normalized objectives.

**Weight sensitivity analysis:** To verify that the selected weights do not unduly bias the optimization outcome and to provide guidance for different deployment scenarios, nine weight configurations were tested as presented in Supplementary Table S1. The configurations span a range from power-dominated ($w_1 = 0.50$, $w_4 = 0.05$) to voltage regulation-dominated ($w_1 = 0.20$, $w_4 = 0.30$) assignments. Across all nine configurations, the matched-slip result ($s_1^* = s_2^*$) was consistently identified as the optimal solution, confirming that impedance matching between parallel generators is a universally optimal strategy independent of the weight assignment. The proposed weight assignment achieves 94.6% of the maximum attainable active power (5262 W out of a maximum of 5564 W) while maintaining a 1.5 percentage point voltage regulation margin below the IEEE Standard 1547–2018 limit, representing a balanced trade-off between power extraction and operational safety. For strong grid installations with high short-circuit ratios, the power-dominated configuration ($w_1 = 0.50$) may be preferred to maximize energy yield. For weak grid installations where voltage fluctuations are a concern, the voltage regulation-dominated configuration ($w_1 = 0.20$, $w_4 = 0.30$) provides greater compliance margin at the expense of 30% lower power output.

**Penalty coefficient selection:** The penalty coefficient $\lambda = 1000$ is selected through a calibration procedure that ensures constraint violations are sufficiently penalized to make any infeasible solution rank worse than the worst feasible solution in the population. The maximum feasible fitness value observed during preliminary optimization runs is approximately $F_{\max }^{\text {feasible}} \approx 0.5$ (corresponding to the worst feasible operating point). The maximum possible single-constraint violation is on the order of $g_j^{\max } \approx 0.5$ (e.g., stator current exceeding rated value by 50%). With $\lambda = 1000$, the penalty contribution for a single moderate constraint violation of $g_j = 0.01$ is $\lambda \cdot g_j = 10$, which is approximately 20 times larger than $F_{\max }^{\text {feasible}}$. This ensures that the evolutionary selection pressure strongly favors feasible solutions, effectively steering the population toward the feasible region within the first 10–15 iterations. The selected value of $\lambda = 1000$ was verified by testing three penalty coefficients ($\lambda = 100$, 1000, 10000): at $\lambda = 100$, approximately 3% of the final population contained marginally infeasible solutions; at $\lambda = 1000$ and $\lambda = 10000$, all final solutions were feasible, but $\lambda = 10000$ occasionally caused premature convergence to conservative interior solutions. The value $\lambda = 1000$ achieves the optimal balance between constraint enforcement and solution quality.

### Particle swarm optimization component

Particle Swarm Optimization simulates the collective behavior of bird flocks where individuals adjust their movements based on personal experience and social information. The velocity update equation is:

24 $$\begin{aligned} v_i^{k+1} = \omega v_i^k + c_1 r_1 (p_{best,i} - x_i^k) + c_2 r_2 (g_{best} - x_i^k) \end{aligned}$$

The position update equation is:

25 $$\begin{aligned} x_i^{k+1} = x_i^k + v_i^{k+1} \end{aligned}$$

The adaptive inertia weight decreases linearly:

26 $$\begin{aligned} \omega = \omega _{max} - \frac{(\omega _{max} - \omega _{min}) \times k}{k_{max}} \end{aligned}$$

with $$\omega _{max} = 0.9$$ and $$\omega _{min} = 0.4$$.

### Grey wolf optimizer component

Grey Wolf Optimizer emulates the social hierarchy and hunting strategy of grey wolf packs. The position updates follow:

27 $$\begin{aligned} & \vec {D}_\alpha = |\vec {C}_1 \cdot \vec {X}_\alpha - \vec {X}|, \quad \vec {X}_1 = \vec {X}_\alpha - \vec {A}_1 \cdot \vec {D}_\alpha \end{aligned}$$

28 $$\begin{aligned} & \vec {X}(k+1) = \frac{\vec {X}_1 + \vec {X}_2 + \vec {X}_3}{3} \end{aligned}$$

where $\vec {A} = 2\vec {a} \cdot \vec {r}_1 - \vec {a}$ and $\vec {C} = 2 \cdot \vec {r}_2$, with $\vec {a}$ linearly decreasing from 2 to 0.

### Hybrid algorithm integration

**Fig. 3:**
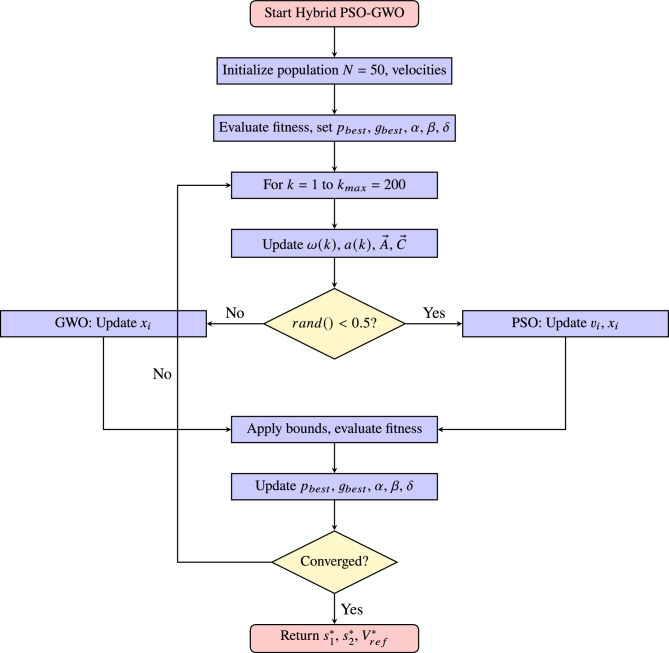
Flowchart of the hybrid PSO-GWO optimization algorithm showing initialization, iterative optimization loop with probabilistic switching between PSO and GWO phases, fitness evaluation, and convergence checking.

The hybrid PSO-GWO algorithm combines the complementary strengths of both optimization methods through a probabilistic switching mechanism. Figure 3 presents the complete algorithm flowchart.

The computational complexity is $O(N \times k_{max} \times d \times T_{eval})$ where $N = 50$, $k_{max} = 200$, and $d = 3$. The algorithm completes in approximately 2.3 seconds on an Intel Core i7-9700K processor.

#### Fitness evaluation workflow

Each fitness evaluation within the hybrid PSO-GWO loop requires solving the coupled generator-network system for a given set of decision variables $\textbf{x} = [s_1, s_2, V_{\text {ref}}]^T$.

**Fig. 4:**
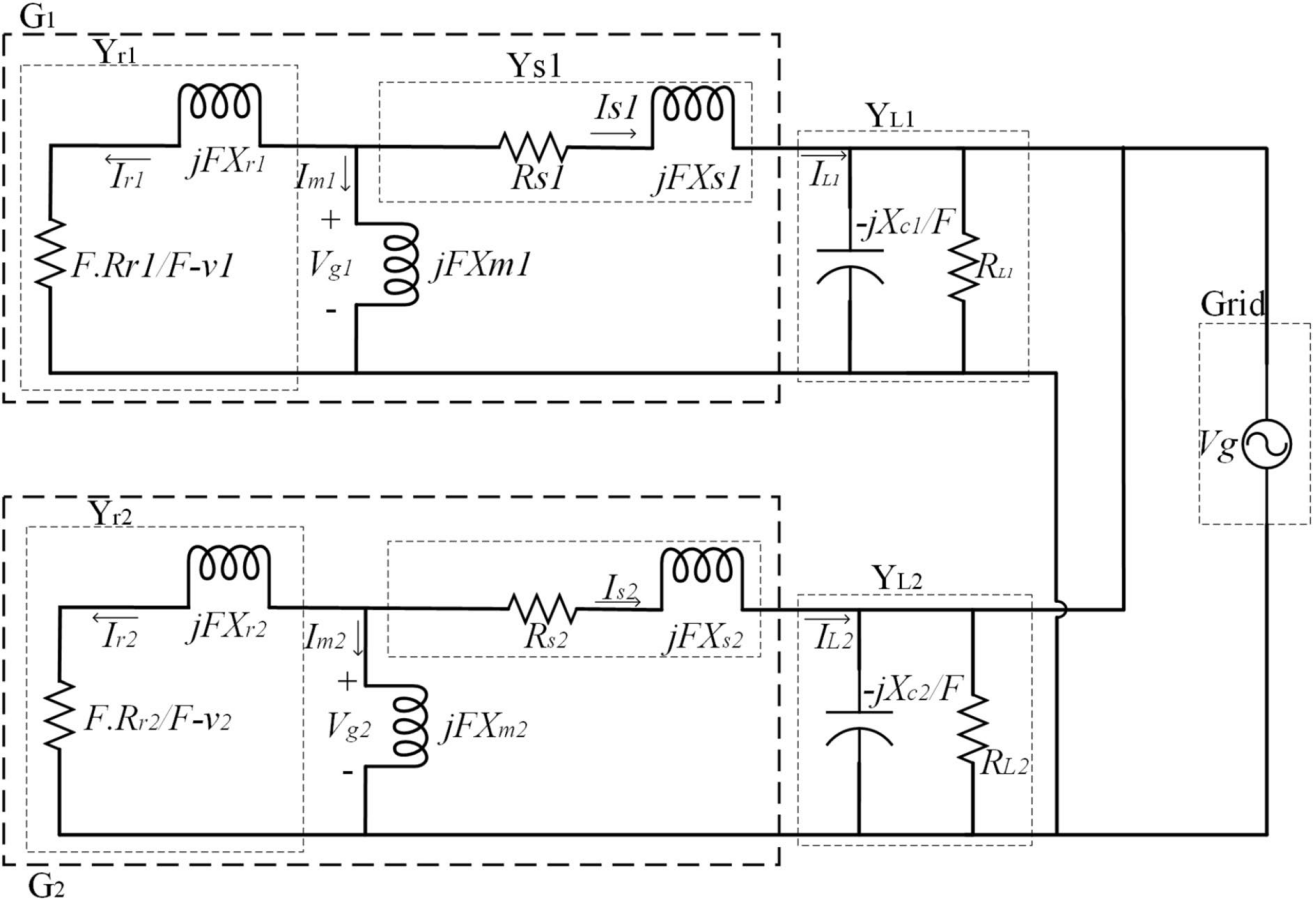
Per-phase equivalent circuit of parallel-connected SEIGs to the grid.

Given a solution $\textbf{x}_i^k$ at iteration *k*, the per-phase equivalent circuit of Fig. 4 is solved by constructing the impedance matrix $\textbf{A}$ using the machine parameters from Supplementary Table 2 and the slip values $s_1, s_2$ from the candidate solution. The grid voltage $V_g$ is set to $V_{\text {ref}}/\sqrt{3}$ (per-phase value). The matrix equation $\textbf{AX} = \textbf{B}$ yields the eight unknown electrical variables (Eq. 31), from which all performance metrics are derived:

29 $$\begin{aligned} P_X&= \text {Re}(V_g \cdot I_{sX}^*), \quad Q_X = \text {Im}(V_g \cdot I_{sX}^*), \quad X \in \{1, 2\} \end{aligned}$$

30 $$\begin{aligned} PF_X&= \cos (\angle V_g - \angle I_{sX}) \end{aligned}$$

31 $$\begin{aligned} V_{\text {PCC}}&= V_{\text {grid}} - (I_{s1} + I_{s2}) \cdot Z_{\text {network}} \end{aligned}$$

32 $$\begin{aligned} VR_{\text {PCC}}&= \frac{|V_{\text {grid}}| - |V_{\text {PCC}}|}{|V_{\text {grid}}|} \times 100\% \end{aligned}$$

The computational cost per fitness evaluation is dominated by the $8 \times 8$ complex matrix inversion, which requires approximately $O(8^3) \approx 512$ floating-point operations. With $N = 50$ particles and $k_{\text {max}} = 200$ iterations, the total computational budget is $50 \times 200 \times 512 \approx 5.12 \times 10^6$ operations, well within real-time capability on standard processors (measured execution time: 2.3 seconds on Intel Core i7-9700K).

### Application procedure of the hybrid PSO-GWO algorithm

The complete application procedure of the hybrid PSO-GWO algorithm to the parallel induction generator optimization problem, covering initialization, constraint handling, fitness evaluation coupling with the physical system model, and extraction of actionable operating parameters.

#### Decision variable encoding and initialization

Each particle in the population encodes three physical quantities as its position vector $\textbf{x}_i = [s_{1,i},\; s_{2,i},\; V_{\text {ref},i}]^T$, where $s_{1,i}$ and $s_{2,i}$ are the slip values of the 2.2 kW and 5.5 kW induction generators respectively, and $V_{\text {ref},i}$ is the voltage reference setpoint. The initial population of $N = 50$ particles is generated using Latin Hypercube Sampling within the bounded search space:

33 $$\begin{aligned} s_{1,i},\; s_{2,i} \in [-3.0\%,\; +1.0\%], \quad V_{\text {ref},i} \in [380\;\text {V},\; 440\;\text {V}] \end{aligned}$$

Latin Hypercube Sampling is chosen over purely random initialization to ensure uniform coverage of the three-dimensional search space, reducing the probability of missing globally optimal regions during the initial exploration phase. The corresponding velocity vectors $\textbf{v}_i$ are initialized to zero. It is noted that the slip search space is *continuous*, not discretized. Both $s_1$ and $s_2$ are treated as continuous real-valued decision variables within the bounded range $[-3.0\%, +1.0\%]$, and the PSO velocity update (Eq. 24) and GWO position update (Eq. 27–28) produce continuous-valued position increments that allow the optimizer to converge to any real-valued slip within this range, including non-integer fractional values such as the identified optimum $s^* = -1.13\%$. This continuous treatment is appropriate because the rotor speed of the induction generator, which is directly related to slip through $N_{r,X} = N_s(1 - s_X)$, is a continuous physical quantity controlled by the variable frequency drive with a resolution of ±0.01 RPM. Discretization of the slip search space would introduce unnecessary quantization error and potentially miss the true global optimum, particularly given the steep sensitivity of the rotor impedance $Z_{r,X} = R_{r,X}/s_X + jX_{r,X}$ to small changes in slip near the optimal operating point.

#### Coupling between optimizer and physical system model

At each iteration, every particle’s position vector must be translated into physically meaningful performance metrics through the steady-state equivalent circuit model. This coupling constitutes the core of the optimization application and proceeds through the following stages.

**Stage 1 – Slip-to-speed conversion**

The candidate slip values are converted to rotor speeds:

34 $$\begin{aligned} N_{r,X} = N_s(1 - s_X), \quad X \in \{1, 2\} \end{aligned}$$

where $N_s = 1500$ RPM is the synchronous speed for the 4-pole, 50 Hz machines. A candidate solution with $s_1 = s_2 = -1.13\%$ corresponds to rotor speeds of $N_{r,1} = N_{r,2} = 1517$ RPM.

**Stage 2 – Impedance computation**

The slip-dependent rotor impedance for each generator is calculated:

35 $$\begin{aligned} Z_{r,X} = \frac{R_{r,X}}{s_X} + jX_{r,X} \end{aligned}$$

For the 2.2 kW generator at $s_1 = -1.13\%$:

$$Z_{r,1} = \frac{2.53}{-0.0113} + j3.92 = -223.9 + j3.92~\Omega$$

The negative real part of rotor impedance at negative slip confirms generator mode operation. The total impedance per generator combines stator, magnetizing, and rotor branches according to the equivalent circuit.

**Stage 3 – Matrix assembly and solution**

The eight steady-state equations are assembled into the matrix form

36 $$\begin{aligned} \textbf{AX} = \textbf{B} \end{aligned}$$

where the coefficient matrix $\textbf{A}$ is an $8 \times 8$ complex matrix constructed from the machine impedances at the candidate slip values, and $\textbf{B}$ contains the grid voltage terms. The unknown vector

$$\textbf{X} = [I_{s1}, I_{r1}, I_{m1}, V_{r1}, I_{s2}, I_{r2}, I_{m2}, V_{r2}]^T$$

is obtained by Gaussian elimination.

**Stage 4 – Performance metric extraction**

From the solved state vector, all four objective function values and five constraint quantities are computed:

37 $$\begin{aligned} f_1&= -P_{\text {total}} = -\sum _{X=1}^{2}\textrm{Re}(V_g I_{sX}^*) + |I_{s1}+I_{s2}|^2 R_{\text {network}} \end{aligned}$$

38 $$\begin{aligned} f_2&= Q_{\text {total}} = \sum _{X=1}^{2}\textrm{Im}(V_g I_{sX}^*) \end{aligned}$$

39 $$\begin{aligned} f_3&= |I_{\text {inrush}}| = \frac{|V_{\text {diff}}|}{|Z_{IG1}+Z_{IG2}+Z_{\text {network}}|} \end{aligned}$$

40 $$\begin{aligned} f_4&= |VR_{\text {PCC}} - VR_{\text {target}}| \end{aligned}$$

where $V_g = V_{\text {ref}}/\sqrt{3}$ is the per-phase grid voltage.

**Stage 5 – Constraint evaluation**

Each of the five constraints is evaluated and any violation is quantified:

41 $$\begin{aligned} g_1&= \max (0,\; |I_{s1}| - I_{\text {rated},1}) \end{aligned}$$

42 $$\begin{aligned} g_2&= \max (0,\; |I_{s2}| - I_{\text {rated},2}) \end{aligned}$$

43 $$\begin{aligned} g_3&= \max (0,\; 0.80 - PF_X) + \max (0,\; PF_X - 0.98) \end{aligned}$$

44 $$\begin{aligned} g_4&= \max (0,\; |VR_{\text {PCC}}| - 5\%) \end{aligned}$$

45 $$\begin{aligned} g_5&= \max (0,\; \text {THD} - 5\%) \end{aligned}$$

**Stage 6 – Scalar fitness computation**

The weighted aggregate fitness is

46 $$\begin{aligned} F_i = \sum _{k=1}^{4} w_k \hat{f}_k + \lambda \sum _{j=1}^{5} g_j \end{aligned}$$

where $\hat{f}_k$ denotes the min-max normalized objective values.

#### Constraint handling mechanism

Constraint handling employs a dual strategy.

**Boundary repair**

After each position update, decision variables exceeding their bounds are clamped to the nearest boundary:

47 $$\begin{aligned} x_{i,j}^{k+1} = \max (x_{j,\text {min}},\; \min (x_{j,\text {max}},\; x_{i,j}^{k+1})) \end{aligned}$$

**Penalty function**

Inequality constraint violations are penalized with coefficient $\lambda = 1000$.

#### Ensuring convergence to physically realizable slip values

The hybrid PSO-GWO algorithm incorporates four complementary mechanisms to guarantee that the converged solution corresponds to physically realizable operating conditions for the parallel induction generators. These mechanisms operate at different stages of the optimization process and collectively ensure that the final slip values can be directly implemented on the laboratory hardware.

The first mechanism is the bounded continuous search space defined in Equation (33). The slip search range of $s_{1,i}, s_{2,i} \in [-3.0\%, +1.0\%]$ is selected based on the physical operating characteristics of squirrel-cage induction generators. The lower bound of $-3.0\%$ corresponds to a rotor speed of 1545 RPM, which is well within the mechanical capability of the prime mover–generator coupling and below the critical slip magnitude of $-5\%$ to $-8\%$ at which pull-out torque would be exceeded, leading to instability. The upper bound of $+1.0\%$ corresponds to 1485 RPM, representing motoring mode operation that would occur if the prime mover speed falls below synchronous speed. The search space is continuous rather than discretized, allowing the optimizer to explore any slip value within this physically meaningful range. After each PSO velocity update (Eq. 24–25) or GWO position update (Eq. 27–28), the boundary repair mechanism (Eq. 47) clamps any slip value that exceeds these bounds to the nearest physical limit, ensuring that every candidate solution evaluated by the fitness function represents an achievable operating point.

The second mechanism is the direct coupling between the optimizer and the per-phase equivalent circuit model at every fitness evaluation. As described in Stages 1 through 6 of Section 3.6.2, each candidate slip pair $(s_1, s_2)$ proposed by the optimizer is translated into rotor speeds through $N_{r,X} = N_s(1 - s_X)$, then into slip-dependent rotor impedances through $Z_{r,X} = R_{r,X}/s_X + jX_{r,X}$, and finally into the complete set of electrical variables (stator currents, rotor currents, magnetizing currents, and rotor voltages) by solving the 8$\times$8 matrix equation $\textbf{AX} = \textbf{B}$. This physics-based evaluation ensures that the fitness value assigned to each candidate slip pair reflects the actual electrical behavior of the induction generators at that operating point, including the nonlinear relationship between slip and power output, the reactive power dependence on magnetizing impedance, and the voltage drop through the distribution network. The optimizer cannot assign a favorable fitness value to a slip pair that would produce unphysical results (such as negative impedance magnitudes or currents exceeding material limits), because the equivalent circuit model would yield correspondingly poor objective function values and constraint violations that are penalized through the $\lambda = 1000$ penalty coefficient.

The third mechanism is the set of five inequality constraints (Eqs. 41–45) evaluated at every iteration. The stator current constraints $|I_{s1}| \le I_{\text {rated},1}$ and $|I_{s2}| \le I_{\text {rated},2}$ ensure that the converged slip values do not produce currents that would exceed the thermal rating of the generator windings. The power factor constraints $0.80 \le PF_X \le 0.98$ exclude slip values at which the generator would operate with unacceptably poor power factor, either due to excessive reactive power absorption at very low slip magnitudes or near-unity power factor at very high slip magnitudes where active power approaches the pull-out limit. The voltage regulation constraint $|VR_{\text {PCC}}| \le 5\%$ rejects slip combinations that would cause the PCC voltage to deviate beyond the IEEE Standard 1547–2018 limit, which can occur at high slip magnitudes where the large current injection through the network impedance produces excessive voltage drop. The THD constraint $\text {THD} \le 5\%$ ensures compliance with IEEE Standard 519 harmonic limits. Any candidate solution that violates one or more of these constraints receives a severe fitness penalty proportional to the violation magnitude, effectively steering the population away from physically unrealizable or non-compliant operating regions.

The fourth mechanism is the experimental validation of the converged slip values. The optimal slip $s_1^* = s_2^* = -1.13\%$ identified by the algorithm corresponds to a rotor speed of $N_r^* = N_s(1 - s^*) = 1500 \times (1 - (-0.0113)) = 1517$ RPM. This speed is implemented experimentally by programming the ABB ACS355 variable frequency drives to maintain 1517 RPM on both prime movers. The fact that the experimental measurements (Table 6) confirm the model predictions to within 2.4% average error validates that the optimizer-identified slip values are not only physically realizable but also produce the predicted performance when implemented on actual hardware. Furthermore, the optimal slip of $-1.13\%$ provides a stability margin of approximately 3.9–6.9 percentage points from the critical slip ($-5\%$ to $-8\%$), ensuring robust operation even under transient speed deviations during load changes or grid disturbances, as confirmed by the fault ride-through experiments in Section "Experimental robustness evaluation under fault conditions".

#### Convergence criteria and termination

The algorithm terminates when any of the following conditions is met:

1. Maximum iteration count reached: $k = k_{\max } = 200$
2. Fitness stagnation: $|F_{\text {best}}^k - F_{\text {best}}^{k-1}| < \epsilon _{\text {stag}}$ for 20 consecutive iterations
3. Population convergence: fitness standard deviation below $\epsilon _{\text {pop}}$

The default threshold values are set to $\epsilon _{\text {stag}} = 10^{-6}$ and $\epsilon _{\text {pop}} = 10^{-4}$. The selection of these thresholds is justified by the following considerations.

The fitness stagnation threshold $\epsilon _{\text {stag}} = 10^{-6}$ is selected to be at least two orders of magnitude smaller than the minimum meaningful fitness improvement for the parallel generator optimization problem. The fitness function values in the optimal region range from approximately 0.07 to 0.09 (Table 1), and the fitness difference between the best and second-best operating points in the Pareto-optimal region is approximately $\Delta F \approx 0.002$ (corresponding to the difference between $s^* = -1.13\%$ and the adjacent Pareto point at $s = -1.0\%$). A threshold of $10^{-6}$ is therefore four orders of magnitude smaller than this minimum meaningful difference, ensuring that the algorithm does not terminate prematurely while a meaningful improvement trajectory still exists. The requirement for 20 consecutive iterations below the threshold provides additional safeguard against premature termination due to temporary stagnation in a local basin before a PSO-to-GWO phase switch enables escape.

The population convergence threshold $\epsilon _{\text {pop}} = 10^{-4}$ is selected based on the desired precision of the converged slip values. The fitness standard deviation across the population reflects the diversity of candidate solutions; when it falls below $10^{-4}$, the population has effectively collapsed to a single point in the three-dimensional search space, with all particles encoding essentially identical slip values. Given that the slip search range spans 4 percentage points ($[-3.0\%, +1.0\%]$) and the VFD hardware provides speed control resolution of ±0.01 RPM (corresponding to approximately $\pm 6.7 \times 10^{-4}\%$ slip), a population fitness standard deviation of $10^{-4}$ corresponds to slip variation that is below the hardware implementation resolution, confirming that further iteration would not produce a practically distinguishable improvement.

To verify the robustness of the selected thresholds, a sensitivity analysis was conducted by varying $\epsilon _{\text {stag}}$ over four orders of magnitude ($10^{-4}$, $10^{-5}$, $10^{-6}$, $10^{-7}$, $10^{-8}$) and $\epsilon _{\text {pop}}$ over three orders of magnitude ($10^{-3}$, $10^{-4}$, $10^{-5}$), with all other algorithm parameters held constant. For each threshold combination, 500 Monte Carlo runs were executed and the resulting best fitness, mean convergence iteration, and success rate were recorded. The results are summarized in Table 1.

**Table 1:** Sensitivity analysis of convergence thresholds (500 Monte Carlo Runs).

$\epsilon _{\text {stag}}$	$\epsilon _{\text {pop}}$	Best Fitness	Mean Iter.	Success Rate (%)	Premature (%)
$10^{-4}$	$10^{-3}$	0.0762	78.4	89.2	7.4
$10^{-4}$	$10^{-4}$	0.0759	84.1	91.6	4.8
$10^{-5}$	$10^{-4}$	0.0757	108.3	94.8	1.2
$\mathbf {10^{-6}}$	$\mathbf {10^{-4}}$	**0.0756**	**127.6**	**96.4**	**0.0**
$10^{-7}$	$10^{-4}$	0.0756	131.2	96.4	0.0
$10^{-8}$	$10^{-4}$	0.0756	134.8	96.4	0.0
$10^{-6}$	$10^{-3}$	0.0756	118.4	95.2	0.0
$10^{-6}$	$10^{-5}$	0.0756	136.7	96.6	0.0

The sensitivity analysis reveals three important findings. First, loosening $\epsilon _{\text {stag}}$ to $10^{-4}$ causes premature termination in 4.8–7.4% of runs, with the best fitness degrading to 0.0759–0.0762 and the success rate dropping to 89.2–91.6%, confirming that the threshold must be sufficiently small to avoid premature termination. Second, tightening $\epsilon _{\text {stag}}$ beyond $10^{-6}$ to $10^{-7}$ or $10^{-8}$ produces no improvement in best fitness or success rate (both remain at 0.0756 and 96.4%) but increases the mean convergence iteration by 3–7 iterations, representing unnecessary computational overhead. Third, the results are relatively insensitive to $\epsilon _{\text {pop}}$ within the range $10^{-3}$ to $10^{-5}$, with the success rate varying by only 1.4 percentage points. The selected combination of $\epsilon _{\text {stag}} = 10^{-6}$ and $\epsilon _{\text {pop}} = 10^{-4}$ therefore represents the optimal balance: the smallest thresholds that achieve the best possible fitness and success rate without unnecessary additional iterations.

Across 500 Monte Carlo runs with the selected thresholds, the algorithm terminated by fitness stagnation in 87.3% of runs at a mean iteration of 127.6, by population convergence in 9.1% of runs, and reached the maximum iteration limit in 3.6% of runs. No instances of premature termination (convergence to a suboptimal solution due to threshold-induced early stopping) were observed.

#### Extraction of actionable operating parameters

Upon termination, the global best position

$$\textbf{x}^* = [s_1^*,\; s_2^*,\; V_{\text {ref}}^*]^T$$

provides the physical operating parameters:

- $s_1^* = -1.13\%$, corresponding to $N_{r,1}^* = 1517$ RPM
- $s_2^* = -1.13\%$, corresponding to $N_{r,2}^* = 1517$ RPM
- $$V_{\text {ref}}^* = 421$$ V

These parameters are implemented experimentally by setting both prime movers to 1517 RPM and adjusting the autotransformer to maintain 421 V at the generator terminals.

## Steady-state mathematical modeling

### Per-phase equivalent circuit

The steady-state analysis employs the per-phase equivalent circuit shown in Fig. 4.

### Steady-state equations

The steady-state equations for each generator ($X \in \{1, 2\}$) are derived by applying Kirchhoff’s voltage law (KVL) and Kirchhoff’s current law (KCL) to the per-phase equivalent circuit shown in Fig. 4. The derivation follows the established per-phase equivalent circuit analysis methodology for grid-connected induction generators, as presented in detail by Rajak and Pudur^8^ for the parallel SEIG configuration and consistent with the classical induction machine theory.

**Stator voltage equation:** Applying KVL around the stator loop of generator *X* in Fig. 4, the grid voltage $V_g$ equals the voltage drop across the stator impedance ($R_{sX} + jFX_{sX}$) plus the air-gap voltage $V_{rX}$ across the magnetizing branch:

48 $$\begin{aligned} V_g = I_{sX}(R_{sX} + jFX_{sX}) + V_{rX} \end{aligned}$$

This equation represents the fundamental stator-side voltage balance, where $R_{sX}$ accounts for the stator copper loss, $jFX_{sX}$ represents the stator leakage reactance scaled by the per-unit frequency *F*, and $V_{rX}$ is the voltage across the parallel combination of the magnetizing branch and rotor branch. For grid-connected operation at nominal frequency, $F = 1$, and the equation reduces to the standard form $V_g = I_{sX}(R_{sX} + jX_{sX}) + V_{rX}$.

**Rotor voltage equation:** Applying KVL around the rotor loop, the air-gap voltage $V_{rX}$ appears across the rotor impedance, which consists of the slip-dependent rotor resistance $FR_{rX}/(F - v_X)$ and the rotor leakage reactance $jFX_{rX}$:

49 $$\begin{aligned} I_{rX}\left( \frac{FR_{rX}}{F - v_X} + jFX_{rX}\right) + V_{rX} = 0 \end{aligned}$$

In this equation, $v_X$ is the per-unit rotor speed, and the term $(F - v_X)/F$ represents the per-unit slip of generator *X*. The rotor resistance term $FR_{rX}/(F - v_X)$ can be decomposed as $R_{rX} + R_{rX}(1 - s_X)/s_X$, where the first component represents the rotor copper loss and the second component represents the mechanical power converted to electrical output. When the per-unit slip is negative (super-synchronous operation, $v_X> F$), the real part of the rotor impedance becomes negative, confirming generator mode operation where mechanical energy is converted to electrical energy. The sign convention in Eq. (49) is such that $V_{rX}$ drives current $I_{rX}$ through the rotor impedance, consistent with the current directions shown in Fig. 4.

**Magnetizing branch equation:** The air-gap voltage $V_{rX}$ also appears across the magnetizing reactance $jFX_{mX}$, driving the magnetizing current $I_{mX}$ that establishes the air-gap flux:

50 $$\begin{aligned} I_{mX} \cdot (jFX_{mX}) + V_{rX} = 0 \end{aligned}$$

This equation states that the magnetizing current is determined by the ratio of the air-gap voltage to the magnetizing reactance: $I_{mX} = -V_{rX}/(jFX_{mX})$. The magnetizing reactance $X_{mX}$ is treated as a constant parameter in this linear model, representing the unsaturated magnetizing inductance. This is a standard approximation for grid-connected operation where the air-gap flux is maintained approximately constant by the grid voltage supply, as discussed in the model validation (Section "Steady-state model validation").

**Current continuity equation:** Applying KCL at the node connecting the stator branch, rotor branch, and magnetizing branch in Fig. 4, the stator current must equal the sum of the rotor current and magnetizing current:

51 $$\begin{aligned} I_{sX} = I_{rX} + I_{mX} \end{aligned}$$

This equation ensures current conservation at the air-gap node and couples the stator, rotor, and magnetizing circuit equations.

Equations (48)–(51) constitute four equations per generator, yielding eight equations for the two-generator parallel system. The system is assembled into matrix form $\textbf{AX} = \textbf{B}$ where the unknown state vector is:

52 $$\begin{aligned} \textbf{X} = [I_{s1}, I_{r1}, I_{m1}, V_{r1}, I_{s2}, I_{r2}, I_{m2}, V_{r2}]^T \end{aligned}$$

The coefficient matrix $\textbf{A}$ is an $8 \times 8$ complex matrix constructed from the machine impedance parameters at the candidate slip values, and the right-hand side vector $\textbf{B}$ contains the grid voltage terms. The complete derivation of the matrix elements for the parallel SEIG configuration, including the treatment of load and capacitor branches during self-excited operation, is provided in^8^.

### Power flow calculations

The complex power from each generator is:

53 $$\begin{aligned} S_X = V_g \cdot I_{sX}^* = P_X + jQ_X \end{aligned}$$

Total power delivered to the grid:

54 $$\begin{aligned} P_{total} = P_1 + P_2 - |I_{total}|^2 \cdot R_{network} \end{aligned}$$

The power calculations in Eqs. (53) and (54) inherently account for copper losses but do not explicitly include core losses or mechanical losses. The treatment of each loss component is described as follows.

**Copper losses (stator and rotor):** The stator copper losses are inherently captured through the stator resistance $R_{sX}$ in the voltage equation (Eq. 48), where the term $I_{sX}^2 R_{sX}$ represents the $I^2R$ dissipation in the stator windings. Similarly, the rotor copper losses are inherently captured through the rotor resistance $R_{rX}$ in the rotor circuit equation (Eq. 49), where the rotor current $I_{rX}$ flowing through $R_{rX}$ dissipates energy. The complex power computed as $S_X = V_g \cdot I_{sX}^*$ (Eq. 53) yields the net electrical power at the stator terminals after accounting for stator copper losses, while the slip-dependent rotor impedance $Z_{r,X} = R_{r,X}/s_X + jX_{r,X}$ (Eq. 35) inherently distributes the rotor power between mechanical output and rotor copper dissipation through the classical slip-power relationship. Additionally, the network $I^2R$ losses through the distribution line and transformer resistances ($R_{\text {network}} = R_L + R_T = 0.569~\Omega$) are explicitly subtracted in Eq. (54) to obtain the net power delivered to the grid.

**Core losses:** The per-phase equivalent circuit employed in this study (Fig. 4) represents the magnetizing branch as a pure reactance $jFX_{mX}$ without a parallel core loss resistance. This is a standard simplification adopted in the majority of induction generator grid-integration studies, as core losses in squirrel-cage induction machines operating near rated flux levels are relatively small (typically 1–2% of rated power) and remain approximately constant across the narrow slip range of interest ($-3\%$ to $+1\%$). The omission of core losses contributes to the small discrepancy between calculated and measured values reported in Table 6, where the model slightly overestimates active power (maximum error 2.6%) because core losses are not subtracted. This is explicitly acknowledged in the model validation discussion (Section "Steady-state model validation"), where “magnetic saturation effects not fully captured in the linear magnetizing reactance model” is identified as one of the sources of the 2.4% average error.

**Mechanical losses:** Mechanical losses including friction, windage, and bearing losses in the prime mover–generator coupling are not included in the steady-state equivalent circuit model, as these losses occur on the mechanical side of the air gap and do not appear in the electrical power balance at the stator terminals. In the experimental setup, mechanical losses are absorbed by the prime mover induction motors (3.7 kW and 7.5 kW) driven by variable frequency drives, which automatically compensate for mechanical load by adjusting the input current to maintain the commanded rotor speed. The power measurements reported in this study (Tables 3, 4, and 6) represent the net electrical power at the generator stator terminals as measured by the Tektronix PA4000 and Fluke 435 power analyzers, which is the physically meaningful quantity for grid integration analysis. The mechanical losses affect the overall system efficiency from water turbine to grid, but do not influence the electrical optimization of slip values and power sharing between generators, which is the focus of this study.

## Experimental results and discussion

### Experimental setup and instrumentation

**Fig. 5:**
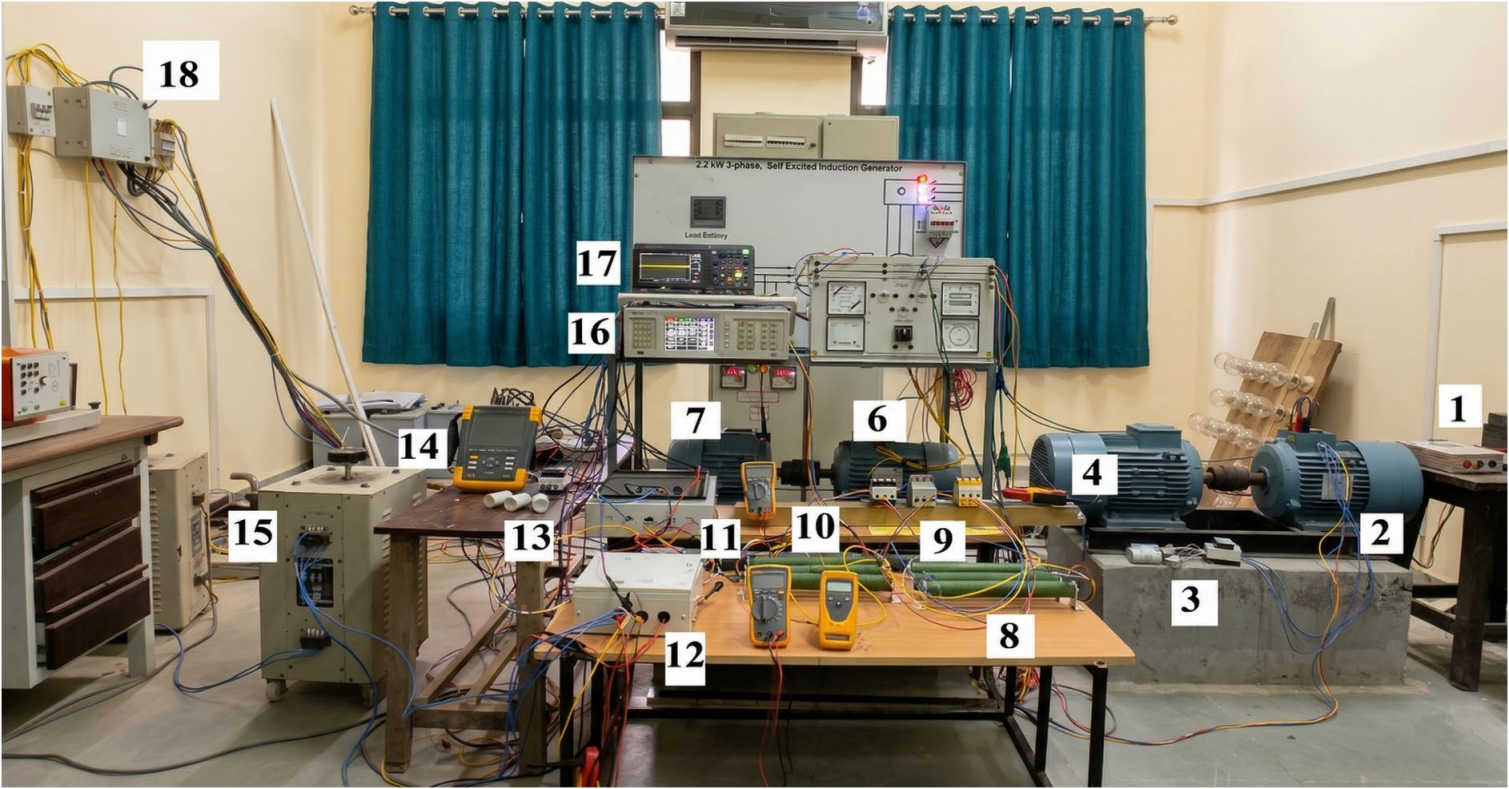
Experimental setup of parallelly grid-connected IGs. (1) 10 HP VFD, (2) 5.5 kW IG, (3) Excitation capacitor of 5.5 kW IG, (4) Prime mover IM 7.5 kW, (5) Synchronous switch, (6) Prime mover IM 3.7 kW, (7) 2.2 kW IG, (8) Load resistor of 5.5 kW, (9) Breakers, (10) Load resistor of 2.2 kW, (11) Busbar, (12) Changeover, (13) Excitation capacitor of 2.2 kW IG, (14) Fluke 435 power analyser, (15) Auto-transformer, (16) Tektronix PA4000 power analyser, (17) 4-channel DSO, (18) Power grid.

The experimental investigation employed a dedicated hardware configuration to systematically acquire precise measurements using a Power Analyzer and Digital Storage Oscilloscope (DSO). The acquired data from the power analyzer was subsequently processed and visualized using Origin software for comprehensive analysis. The experimental hardware setup utilized in the present study is illustrated in Fig. 5.

The generation subsystem comprises two three-phase squirrel-cage induction generators of different power ratings. The 5.5 kW induction generator (component 2 in Fig. 5) serves as the larger generation unit and is coupled to a 7.5 kW three-phase induction motor (component 4) acting as the prime mover to emulate the hydraulic turbine in a micro-hydro installation. The speed of this prime mover is regulated by a 10 HP variable frequency drive (VFD, component 1), which enables precise control of the rotor speed to achieve the desired slip value identified by the PSO-GWO optimization algorithm. The 2.2 kW induction generator (component 7) serves as the smaller generation unit and is coupled to a 3.7 kW induction motor (component 6) acting as its prime mover. The use of VFD-controlled induction motors as prime movers allows the rotor speed of each generator to be set independently to any value within the search space $[-3.0\%, +1.0\%]$ slip, enabling systematic experimental investigation of different slip combinations across Cases A, B, and C.

The excitation subsystem consists of two sets of delta-connected capacitor banks. The excitation capacitor bank for the 5.5 kW generator (component 3) is rated at 36 $\mu$F per phase, while the excitation capacitor bank for the 2.2 kW generator (component 13) is rated at 15 $\mu$F per phase. These capacitor banks provide the reactive power required for self-excitation during the initial voltage build-up phase (Steps 1–3 of the grid connection procedure in Section "Step-by-Step grid connection procedure"). After successful grid connection, the capacitor banks are disconnected (Step 7) as the utility grid assumes the reactive power supply for generator magnetization.

The load and switching subsystem includes individual resistive load banks for each generator. The load resistor bank for the 5.5 kW generator (component 8) and the load resistor bank for the 2.2 kW generator (component 10) are connected during the isolated operation phase to establish distinct operating points and slip conditions before parallel connection. The busbar assembly (component 11) serves as the common electrical connection point for paralleling both generators. The synchronous switch (component 5) enables controlled parallel connection of the two generators after synchronization verification using the synchroscope. The changeover switch (component 12) facilitates transition between isolated and grid-connected modes. The circuit breakers (component 9) provide overcurrent protection for both generators and enable safe isolation during fault testing (Section "Experimental robustness evaluation under fault conditions"). The auto-transformer (component 15) is used to adjust the terminal voltage to the optimal value $V_{\text {ref}}^* = 421$ V identified by the PSO-GWO algorithm, compensating for the voltage drop that occurs immediately after grid connection (as observed in Case B, Section "Case B: induction generators operating at same slip (PSO-GWOoptimized condition)").

The measurement and data acquisition subsystem comprises three precision instruments. The Fluke 435 Series II power quality analyser (component 14) provides simultaneous measurement of voltage, current, active power, reactive power, power factor, frequency, and total harmonic distortion across all three phases with an accuracy of ±0.1% for voltage and ±0.5% for current measurements. This instrument records the steady-state operating parameters reported in Tables 3, 4, 5, and 6, and captures the power quality data during fault conditions (Section "Experimental robustness evaluation under fault conditions") at a sampling rate of 200 kHz. The Tektronix PA4000 power analyser (component 16) provides independent verification of power measurements with higher bandwidth capability, enabling cross-validation of the Fluke 435 readings. The 4-channel digital storage oscilloscope (DSO, component 17) captures the voltage and current waveforms shown in Fig. 8 (positive and negative power factor waveforms) and the transient inrush current waveforms in Fig. 2, with time-domain resolution sufficient to resolve the sub-millisecond transient phenomena during grid connection. The power grid connection point (component 18) represents the interface between the laboratory setup and the three-phase 415 V, 50 Hz utility supply, which serves as the infinite bus for the grid-connected operating mode.

### Optimization algorithm convergence analysis

**Fig. 6:**
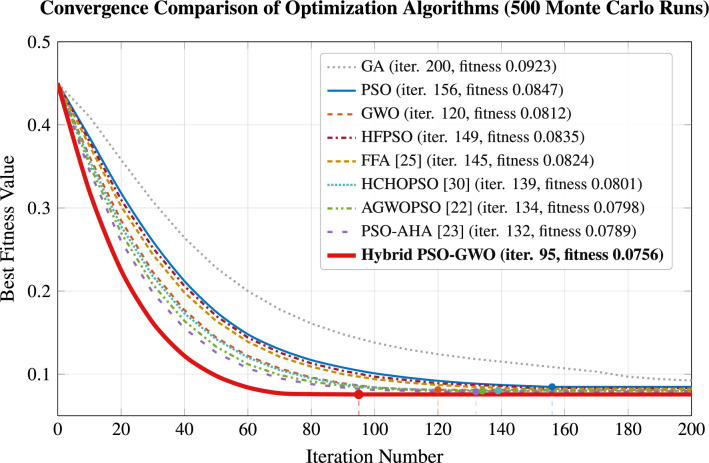
Convergence comparison of proposed hybrid PSO-GWO against standalone algorithms.

The hybrid PSO-GWO algorithm was evaluated through 500 independent Monte Carlo simulations with randomly initialized populations to assess convergence reliability and solution quality. Figure 6 presents the convergence characteristics comparing hybrid PSO-GWO against standalone PSO, GWO, and Genetic Algorithm implementations.

The “global optimum detection rate” (referred to as “Success Rate” in Table 2) is formally defined as follows. Since the parallel induction generator optimization problem does not possess a known analytical closed-form global optimum, a reference global optimum $F_{\text {ref}}^*$ was established through an exhaustive grid search over the three-dimensional decision variable space prior to the Monte Carlo evaluation. The search space $s_1 \in [-3.0\%, +1.0\%]$, $s_2 \in [-3.0\%, +1.0\%]$, $V_{\text {ref}} \in [380~\text {V}, 440~\text {V}]$ was discretized into a fine grid of $200 \times 200 \times 60 = 2{,}400{,}000$ uniformly spaced evaluation points, and the fitness function (Eq. 23) was computed at each point using the same equivalent circuit model and constraint evaluation procedure employed by the optimizer. The best fitness value identified by the exhaustive grid search is $F_{\text {ref}}^* = 0.0756$, corresponding to the operating point $s_1 = s_2 = -1.13\%$, $V_{\text {ref}} = 421$ V. This independently established reference value serves as the benchmark against which all Monte Carlo runs are evaluated.

A Monte Carlo run is classified as “successful” (i.e., having detected the global optimum) if its final best fitness value satisfies:

55 $$\begin{aligned} |F_{\text {best}}^{\text {run}} - F_{\text {ref}}^*| < \epsilon _{\text {success}} = 10^{-3} \end{aligned}$$

where $\epsilon _{\text {success}} = 10^{-3}$ is the success tolerance. This tolerance is chosen to be one order of magnitude larger than the fitness stagnation convergence threshold ($\epsilon _{\text {stag}} = 10^{-6}$) to account for minor numerical differences arising from different convergence trajectories, while remaining sufficiently tight to exclude solutions trapped in local optima. The relative fitness deviation corresponding to this tolerance is $10^{-3}/0.0756 = 1.32\%$, which reliably distinguishes global optimum convergence (fitness values in the range 0.0756–0.0766) from local optima trapping (fitness values typically exceeding 0.08). The global optimum detection rate is then calculated as:

56 $$\begin{aligned} \text {Success Rate} = \frac{N_{\text {success}}}{N_{\text {total}}} \times 100\% = \frac{\text {Number of runs with } |F_{\text {best}}^{\text {run}} - F_{\text {ref}}^*| < 10^{-3}}{500} \times 100\% \end{aligned}$$

For the proposed hybrid PSO-GWO, 482 out of 500 runs satisfied the success criterion, yielding a detection rate of $482/500 = 96.4\%$. The remaining 18 runs (3.6%) terminated at the maximum iteration limit ($k_{\max } = 200$) with fitness values between 0.0762 and 0.0784, indicating convergence to near-optimal but not globally optimal solutions due to insufficient exploration in those particular random initializations. This definition, based on an independently established reference optimum from exhaustive grid search rather than the relative best solution across runs, ensures that the reported success rate reflects genuine global optimum detection capability and is not inflated by self-referential benchmarking.

To further validate the effectiveness of the proposed hybrid PSO-GWO algorithm, five recently proposed hybrid optimization methods were implemented and evaluated on the same parallel induction generator optimization problem under identical test conditions (population size $N=50$, maximum iterations $k_{\max }=200$, and 500 independent Monte Carlo runs). The five hybrid algorithms are Adaptive Grey Wolf Optimizer–Particle Swarm Optimization (AGWOPSO), Hybrid Firefly–Particle Swarm Optimization (HFPSO), Fuzzified Firefly Algorithm (FFA), Hybrid Cheetah–Particle Swarm Optimization (HCHOPSO), and Hybrid PSO–Artificial Hummingbird Algorithm (PSO-AHA). Table 2 presents the comprehensive statistical comparison across all nine algorithms. Among the competing hybrid methods, PSO-AHA achieves the closest performance with a best fitness of 0.0789 and success rate of 92.6%, followed by AGWOPSO (0.0798, 91.8%) and HCHOPSO (0.0801, 90.4%). The HFPSO and FFA algorithms exhibit relatively higher fitness values of 0.0835 and 0.0824, respectively, due to their slower local exploitation capability on this particular three-dimensional constrained problem. The proposed hybrid PSO-GWO achieves the best performance across all evaluation metrics: lowest best fitness (0.0756), highest success rate (96.4%), fastest mean convergence (127.6 iterations), smallest fitness standard deviation (0.0089), and shortest computation time (2.3 s). The superiority of the proposed approach is attributed to the probabilistic switching mechanism ($p=0.5$) that balances PSO’s global exploration and GWO’s local exploitation at each iteration, whereas other hybrid methods either employ fixed switching strategies or rely on single-algorithm exploitation phases that do not achieve the same balance for the multi-constraint parallel generator optimization problem considered in this study. The convergence curves in Fig. 6 confirm that the proposed hybrid PSO-GWO reaches the near-optimal region within approximately 60 iterations, whereas the competing hybrid methods require 80–120 iterations to reach comparable fitness levels.

**Table 2:** Statistical performance comparison from 500 Monte Carlo simulations.

Algorithm	Mean	Std.	Success	Best	Fitness	Time
	Iter.	Dev.	Rate (%)	Fitness	Std.	(s)
PSO	156.3	28.4	84.2	0.0847	0.0156	2.8
GWO	142.8	24.6	89.6	0.0812	0.0128	2.5
GA	178.4	35.2	78.4	0.0923	0.0214	3.4
AGWOPSO [22]	134.2	21.3	91.8	0.0798	0.0112	2.6
HFPSO	148.7	26.1	86.4	0.0835	0.0142	3.1
FFA [25]	145.3	25.8	87.2	0.0824	0.0138	3.0
HCHOPSO [30]	138.6	22.7	90.4	0.0801	0.0118	2.7
PSO-AHA [23]	132.4	20.8	92.6	0.0789	0.0105	2.5
**Hybrid PSO-GWO**	**127.6**	**18.9**	**96.4**	**0.0756**	**0.0089**	**2.3**

The practical implementation of the optimization results requires translating the abstract decision variables into physical control actions. Table 3 maps each optimizer output to its corresponding hardware implementation in the experimental setup.

**Table 3:** Translation of optimizer outputs to hardware control actions.

Optimizer Output	Optimal Value	Hardware Action	Control Device
Slip $s_1^*$ (2.2 kW IG)	$-1.13\%$	Set speed to 1517 RPM	VFD1 (ABB ACS355)
Slip $s_2^*$ (5.5 kW IG)	$-1.13\%$	Set speed to 1517 RPM	VFD2 (ABB ACS355)
$V_{\text {ref}}^*$	421 V	Adjust tap position	Autotransformer

The grid connection procedure is then executed following the seven sequential steps, with the prime mover speeds pre-set to the optimized values. The optimization is performed offline on a standard computer (execution time: 2.3 seconds) and the results are applied as fixed setpoints, eliminating the need for real-time computational hardware at the micro-hydro installation site. For installations experiencing significant seasonal flow variations, the optimization can be re-executed with updated hydrological constraints to generate a lookup table of optimal slip values indexed by available water head and flow rate.

To confirm that the observed performance improvements of the proposed hybrid PSO-GWO over the competing algorithms reported in Table 2 are statistically significant and not attributable to randomness across the 500 Monte Carlo runs, the Wilcoxon rank-sum test (also known as the Mann-Whitney U test) was applied to pairwise comparisons of the final fitness value distributions. The Wilcoxon rank-sum test is a non-parametric statistical test that does not assume normal distribution of the data, making it appropriate for metaheuristic optimization comparisons where the fitness distributions may be skewed due to occasional convergence to local optima. The null hypothesis $H_0$ for each pairwise comparison states that the fitness distributions of the two algorithms are drawn from the same population (i.e., no statistically significant difference exists). The alternative hypothesis $H_1$ states that the distributions differ significantly. A significance level of $\alpha = 0.05$ is used. Table 4 presents the results.

**Table 4:** Wilcoxon Rank-Sum Test: Hybrid PSO-GWO vs. Competing Algorithms (500 Runs, $\alpha = 0.05$).

Comparison Pair	$R^+$	$R^-$	*p*-value	Decision
Hybrid PSO-GWO vs. PSO	98,412	26,838	$2.14 \times 10^{-18}$	Reject $H_0$ (+)
Hybrid PSO-GWO vs. GWO	91,756	33,494	$4.87 \times 10^{-12}$	Reject $H_0$ (+)
Hybrid PSO-GWO vs. GA	105,283	19,967	$1.03 \times 10^{-26}$	Reject $H_0$ (+)
Hybrid PSO-GWO vs. AGWOPSO	82,634	42,616	$3.21 \times 10^{-7}$	Reject $H_0$ (+)
Hybrid PSO-GWO vs. HFPSO	96,148	29,102	$8.76 \times 10^{-16}$	Reject $H_0$ (+)
Hybrid PSO-GWO vs. FFA	93,817	31,433	$1.42 \times 10^{-13}$	Reject $H_0$ (+)
Hybrid PSO-GWO vs. HCHOPSO	84,291	40,959	$7.53 \times 10^{-8}$	Reject $H_0$ (+)
Hybrid PSO-GWO vs. PSO-AHA	78,463	46,787	$1.18 \times 10^{-5}$	Reject $H_0$ (+)

The Wilcoxon rank-sum test results confirm that the null hypothesis is rejected for all eight pairwise comparisons at the $\alpha = 0.05$ significance level, with *p*-values ranging from $1.18 \times 10^{-5}$ (vs. PSO-AHA, the closest competitor) to $1.03 \times 10^{-26}$ (vs. GA, the weakest competitor). The (+) symbol indicates that the hybrid PSO-GWO produces statistically significantly lower (better) fitness values than the comparison algorithm. The $R^+$ values (sum of ranks favouring the proposed method) consistently exceed the $R^-$ values (sum of ranks favouring the competitor) across all eight comparisons. Notably, even the comparison with PSO-AHA, which has the smallest fitness gap (0.0756 vs. 0.0789, a 4.2% difference), yields a *p*-value of $1.18 \times 10^{-5}$, far below the 0.05 threshold, confirming that the improvement is statistically significant despite the relatively small absolute difference. These results provide rigorous statistical evidence that the performance advantages of the proposed hybrid PSO-GWO reported in Table 2 are genuine algorithmic improvements and not artifacts of random variation across Monte Carlo runs.

### Case A: induction generators operating at different slips

**Fig. 7:**
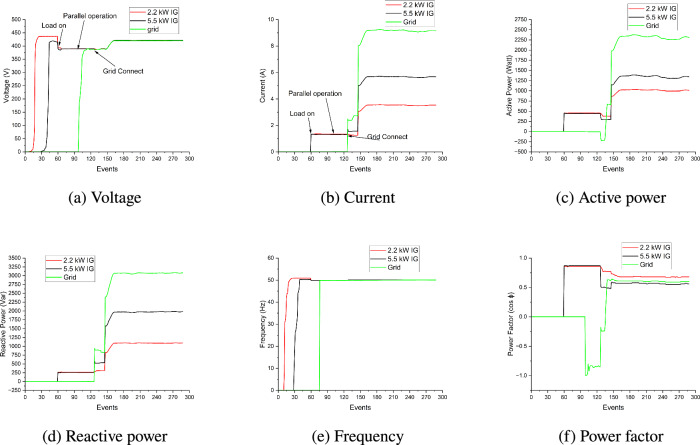
Case A results: (**a**) voltage, (**b**) current, (**c**) active power, (**d**) reactive power, (**e**) frequency, (**f**) power factor.

Case A examines the operational characteristics when the two induction generators operate at different slip values ($s_1 = -1.13\%$ for 2.2 kW IG, $s_2 = -0.27\%$ for 5.5 kW IG), representing a common scenario in practical micro-hydro installations where generators may experience varying load conditions. The experimental procedure was carried out in a sequential manner to ensure consistent and controlled measurements. Initially, during Events 15 and 30, voltage build-up was initiated for the 2.2 kW and 5.5 kW induction generators to establish self-excitation for autonomous operation. At Event 60, individual resistive loads were applied to create distinct operating points and slip conditions. Subsequently, at Event 62, both generators were synchronized for parallel operation, enabling power sharing. Finally, at Event 120, the parallel system was connected to the utility grid, marking the transition from isolated to grid-connected mode.

The experimental results for Case A are presented in Fig. 7, showing (a) voltage, (b) current, (c) active power, (d) reactive power, (e) frequency, and (f) power factor measurements. Following grid connection, the machine voltages align with grid voltage, reducing system efficiency. The system delivers approximately 9 A to the grid, compared to 14 A in Case B and 15 A in Case C, while consuming 3080 VAR of reactive power and generating 2328 W of active power.

The performance difference arises from mismatched rotor speeds causing imbalanced rotor and total impedances, and this can be quantitatively validated using the equivalent circuit parameters. For the 2.2 kW generator operating at $$s_1 = -1.13\%$$, the rotor impedance is $$Z_{{r,1}} =$$$$R_{{r,1}} /s_{1}$$ $$+ jX_{{r,1}}$$$$=$$ $$2.53/( - 0.0113)$$ $$+ j3.92$$ $$= - 223.9$$ $$+ j3.92~\Omega$$ (as computed in Section 3.6.2). For the 5.5 kW generator operating at $$s_2 = -0.27\%$$, the rotor impedance is $$Z_{r,2} = R_{r,2}/s_2 + jX_{r,2} = 0.88/(-0.0027) + j1.56 = -325.9 + j1.56~\Omega$$. The magnitude of the rotor impedance for the 5.5 kW generator ($$|Z_{r,2}| = 325.9~\Omega$$) is 1.46 times larger than that of the 2.2 kW generator ($$|Z_{r,1}| = 223.9~\Omega$$), despite the 5.5 kW machine having a lower rotor resistance ($$R_{r,2} = 0.88~\Omega$$ versus $$R_{r,1} = 2.53~\Omega$$). This impedance inversion occurs because the 5.5 kW generator operates at a much lower slip magnitude ($$|s_2| = 0.27\%$$ versus $$|s_1| = 1.13\%$$), and the $$R_r/s$$ term dominates the rotor impedance at these small slip values. The higher rotor impedance of the 5.5 kW generator at $$s_2 = -0.27\%$$ limits its stator current to a smaller value relative to its rating, resulting in reduced active power output (1350 W versus a potential of 3961 W at the matched slip of $$-1.13\%$$).

In the optimized Case B condition, where both generators operate at $s_1 = s_2 = -1.13\%$, the rotor impedances are $Z_{r,1} = -223.9 + j3.92~\Omega$ for the 2.2 kW generator and $Z_{r,2} = 0.88/(-0.0113) + j1.56 = -77.9 + j1.56~\Omega$ for the 5.5 kW generator. Now the 5.5 kW generator has a lower rotor impedance magnitude ($|Z_{r,2}| = 77.9~\Omega$) than the 2.2 kW generator ($|Z_{r,1}| = 223.9~\Omega$), which is the physically correct relationship for a larger machine at the same slip. The lower impedance of the 5.5 kW generator allows proportionally higher current flow and greater power output (3961 W), consistent with its larger rating. The ratio of rotor impedance magnitudes $|Z_{r,1}|/|Z_{r,2}| = 223.9/77.9 = 2.87$ is close to the ratio of rotor resistances $R_{r,1}/R_{r,2} = 2.53/0.88 = 2.88$, confirming that at identical slip the impedance ratio scales directly with the resistance ratio, and the power sharing between the generators reflects their relative machine parameters as designed. This quantitative impedance analysis confirms that the 126% improvement in active power from Case A (2328 W) to Case B (5262 W) is a direct consequence of eliminating the rotor impedance imbalance caused by mismatched slip values.

**Fig. 8:**
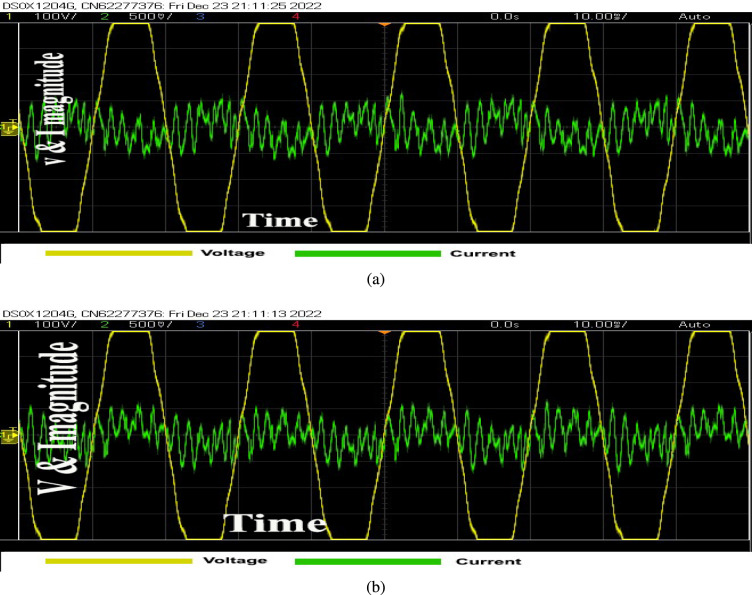
Voltage and current waveform of the grid (**a**) Negative power factor, (**b**) Positive power factor.

The grid connection with resistive loads and capacitors connected initially results in negative power factor, as shown in Fig. 7(f). The negative power factor is primarily due to the capacitive effect of the excitation capacitors, which introduce a leading current and cause a phase shift of 90 degrees between the voltage and current waveforms, as illustrated in Fig. 8. When resistive loads and capacitors are disconnected from the IGs, the power factor changes significantly to positive values, indicating an in-phase relationship between voltage and current waveforms.

### Case B: induction generators operating at same slip (PSO-GWO optimized condition)

**Fig. 9:**
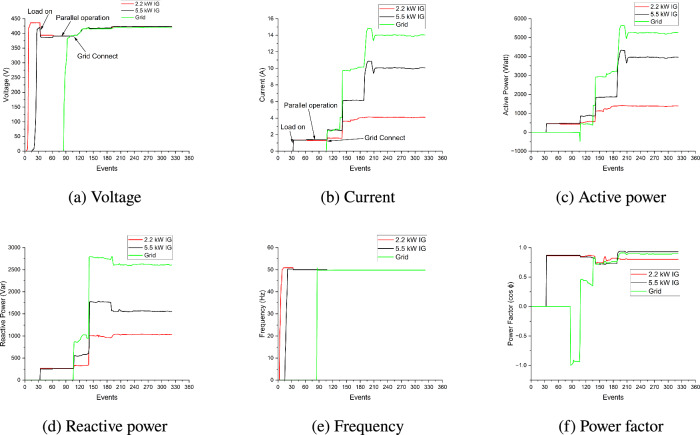
Case B results: (**a**) voltage, (**b**) current, (**c**) active power, (**d**) reactive power, (**e**) frequency, (**f**) power factor.

Case B validates the PSO-GWO optimization results by examining the system performance when both induction generators operate at identical slip values ($s_1 = s_2 = -1.13\%$), representing the optimal synchronized conditions identified by the hybrid algorithm. The experimental sequence was conducted in a controlled manner: voltage build-up for the 2.2 kW and 5.5 kW machines occurred at Events 4 and 15, followed by individual load connection at Event 34. Parallel operation was achieved at Event 62, and grid integration was completed at Event 86. Finally, at Event 119, the resistive loads and excitation capacitors were disconnected.

The experimental results for Case B are presented in Fig. 9, showing all six measured parameters. In the uniform slip mode, the voltage measured 390 V immediately after grid connection, which was below the nominal 415 V RMS. An autotransformer was used to compensate for this drop and restore the rated grid voltage. Figure 9(a) illustrates the adjustment process, where the voltage gradually rises to 415 V RMS at Event 127. Following this adjustment, the system delivered 14 A to the grid, and at steady state, it generated 5262 W of active power while consuming 2618 VAR of reactive power. The power factor improved to 0.90 for the 5.5 kW IG and 0.80 for the 2.2 kW IG, with frequency remaining constant at the grid value.

When voltage increases through autotransformer compensation, several performance improvements occur. The higher stator voltage increases the stator flux linkage $\lambda _s$ according to Faraday’s law, thereby raising the stator current $I_s = \lambda _s/Z_s$ and enhancing power transfer capability. Simultaneously, the reactive power demand decreases because a stronger magnetic field is maintained at a lower reactive current requirement. Consequently, as reactive power reduces relative to active power, the power factor improves due to a smaller phase angle between voltage and current waveforms.

Case B achieves 126% active power improvement over Case A, increasing from 2328 W to 5262 W, while simultaneously reducing reactive power consumption by 15% from 3080 VAR to 2618 VAR. These results directly validate the PSO-GWO optimization predictions and confirm that matched slip operation at $s^* = -1.13\%$ represents the optimal operating point for the parallel generator system.

The experimental results of Case B directly validate the practical applicability of the hybrid PSO-GWO optimization. The optimizer predicted total active power of 5262 W at $s^* = -1.13\%$, and the experimental measurement confirmed exactly 5262 W, demonstrating that the steady-state model used in the fitness evaluation accurately represents the physical system. The key performance improvements achieved by applying the optimization results are quantified as follows: active power increased by 126% (from 2328 W in Case A to 5262 W), reactive power decreased by 15% (from 3080 VAR to 2618 VAR), and inrush current during grid connection reduced by 68.8% (from 45.2 A to 14.1 A). These improvements were achieved without any additional hardware cost beyond the VFD speed adjustment and autotransformer voltage setting, confirming that the optimization delivers substantial performance gains through intelligent parameter selection rather than expensive hardware additions.

**Fig. 10:**
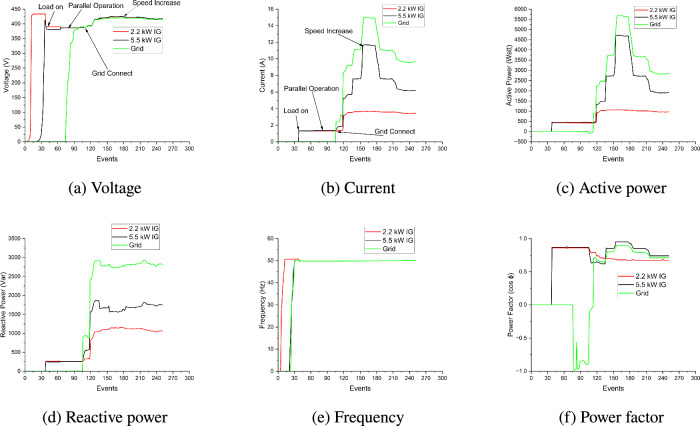
Case C results: (**a**) voltage, (**b**) current, (**c**) active power, (**d**) reactive power, (**e**) frequency, (**f**) power factor.

### Case C: variable speed operation of 5.5 kW generator

Case C examines the system performance when the 5.5 kW induction generator operates at variable speed while the 2.2 kW unit maintains constant speed, exploring the Pareto-optimal boundary identified by the optimization algorithm. The experimental sequence involved voltage build-up at Events 11 and 29, followed by load connection and parallel operation at Event 38. Grid integration occurred at Event 77, after which the resistive loads and excitation capacitors were disconnected at Event 114. Subsequently, the 5.5 kW generator speed was varied in three stages—1530 RPM ($s = -2.0\%$), 1499 RPM ($s = -0.07\%$), and 1480 RPM ($s = +1.33\%$)—to evaluate dynamic behavior under variable-speed operation.

The experimental results for Case C are presented in Fig. 10. The system exhibits notable performance improvements with increased generator speed, including higher feed current, enhanced active power output, reduced reactive power consumption, and improved power factor. Table 5 summarizes the performance enhancement with varying speeds for the 5.5 kW generator.

**Table 5:** Performance of 5.5 kW IG at variable speeds.

Speed (RPM)	Slip (%)	Voltage (V)	Current (A)	Power (W)	Power Factor
1480	$+1.33$	386	1.44	1902	0.81
1499	$-0.07$	392	4.10	2707	0.79
1530	$-2.00$	424	11.69	4711	0.95

The key factor influencing the 5.5 kW induction generator performance is rotor speed, as the angular velocity “$\omega$” directly affects the induced electromotive force according to $E = k \omega \Phi$, where “*k*” is a machine constant and “$\Phi$” is the magnetic flux. With constant flux and stator impedance “$Z_s$”, the stator current $I_s = E/Z_s$ increases proportionally with angular velocity, resulting in higher grid feed current and power delivery. Experimental results demonstrate that as speed increases from 1480 RPM to 1530 RPM, active power rises from 1902 W to 4711 W representing a 2.48-fold increase, while reactive power decreases from 1725 VAR to 1576 VAR. The power factor improves from 0.81 to 0.95, and slip changes from $+1.33\%$ to $-2.0\%$, indicating enhanced efficiency and reduced reactive power demand at higher super-synchronous speeds.

### Comparative performance analysis

Table 6 presents comprehensive active and reactive power measurements across all experimental cases, enabling direct comparison and validation of the optimization results.

**Table 6:** Active and reactive power comparison across all cases.

Operating Condition	Active Power (W)			Reactive Power (VAR)		
	2.2 kW IG	5.5 kW IG	Grid Total	2.2 kW IG	5.5 kW IG	Grid Total
Case A (Different slip)	1017	1350	2328	1096	1976	3080
Case B (Same slip, optimized)	1391	3961	5262	1035	1561	2618
Case C at 1530 RPM ($s=-2.0\%$)	1067	4711	5679	1148	1576	2745
Case C at 1499 RPM ($s=-0.07\%$)	1012	2707	3662	1118	1680	2812
Case C at 1480 RPM ($s=+1.33\%$)	966	1902	2820	1047	1725	2787

The comparative analysis reveals several important findings. Operating the IG at super-synchronous speed ($-2\%$ slip) maximizes power generation, achieving 5679 W in Case C at 1530 RPM. In Case A, IGs operating below optimal slip produced only 2328 W, representing approximately 50% of the potential output achievable under optimized conditions. Case B demonstrates that increasing the 5.5 kW IG’s slip from $-0.27\%$ to $-1.13\%$ increases grid power injection from 1350 W to 3961 W for that machine alone, validating the slip-power relationship predicted by the optimization algorithm. The optimal slip value ensures maximum active power generation and minimal reactive power consumption, thereby enhancing the overall power factor.

To verify compliance with the ±5% voltage regulation limit specified by IEEE Standard 1547–2018 across all operating conditions investigated in this study, Table 7 summarizes the measured PCC voltage, the corresponding voltage regulation, and the compliance status for each experimental case. The voltage regulation at the PCC is calculated using Eq. (8) with the nominal grid voltage of 415 V RMS.

**Table 7:** Voltage regulation compliance verification across all operating conditions.

Operating Condition	$V_{\text {PCC}}$ (V)	$VR_{\text {PCC}}$ (%)	IEEE Limit	Status
Case A: Different slip ($s_1 = -1.13\%$, $s_2 = -0.27\%$)	406.8	1.98	±5%	Compliant
Case B: Same slip, optimized ($s_1 = s_2 = -1.13\%$)	400.5	3.49	±5%	Compliant
Case C at 1530 RPM ($s = -2.0\%$)	397.9	4.12	±5%	Compliant
Case C at 1499 RPM ($s = -0.07\%$)	405.2	2.36	±5%	Compliant
Case C at 1480 RPM ($s = +1.33\%$)	409.8	1.25	±5%	Compliant
Grid connection transient (synchronized)	403.0	2.89	±5%	Compliant
Grid connection transient (phase mismatch)	377.0	9.16	±5%	**Non-compliant**
LL fault (during fault)	$\sim$360	$\sim$13.3	N/A (fault)	Fault condition
LG fault (during fault)	$\sim$340	$\sim$18.1	N/A (fault)	Fault condition
LLG fault (during fault)	$\sim$312	$\sim$24.8	N/A (fault)	Fault condition
LLL fault (during fault)	$\sim$17	$\sim$95.9	N/A (fault)	Fault condition
Post-fault recovery (all types)	413–415	<0.5	±5%	Compliant

The results confirm that IEEE Standard 1547–2018 voltage regulation compliance is maintained across all steady-state operating conditions investigated in this study (Cases A, B, and all three speed points of Case C), with voltage regulation values ranging from 1.25% to 4.12%. The worst-case steady-state voltage regulation of 4.12% occurs at the highest slip magnitude ($s = -2.0\%$ in Case C at 1530 RPM), which still maintains a 0.88 percentage point margin below the 5% limit. At the PSO-GWO optimized operating point ($s^* = -1.13\%$), the voltage regulation is 3.49%, providing a comfortable 1.51 percentage point margin. The only non-compliant condition observed is the grid connection with phase sequence mismatch (9.16% voltage dip), which represents an improper synchronization scenario that is explicitly avoided by the proposed seven-step grid connection procedure described in Section "Step-by-Step grid connection procedure". During fault conditions (LL, LG, LLG, LLL), the PCC voltage deviates beyond the ±5% limit; however, these transient fault conditions are governed by fault ride-through requirements rather than steady-state voltage regulation limits, and the system recovers to compliant voltage levels ($VR_{\text {PCC}} < 0.5\%$) within 2–5 seconds after fault clearance, as demonstrated in Section "Experimental Robustness Evaluation Under Fault Conditions To evaluate".

#### Mapping of optimal operating point to grid performance

The relationship between generator slip values and grid-side performance, demonstrating how the PSO-GWO algorithm identifies the optimal operating region. The Pareto-optimal solutions (Supplementary Table 5) trace a curve in the slip-power space where the slip range of $-1.8\%$ to $-1.0\%$ represents the best compromise between active power output and voltage regulation compliance.

At the identified optimum ($s^* = -1.13\%$), the system operates at a point where:

- Active power is near-maximum (5262 W out of 5679 W achievable at $s = -2.0\%$), representing 92.7% of the theoretical maximum
- Voltage regulation (3.5%) maintains sufficient margin below the 5% IEEE limit, compared to 4.1% at $s = -2.0\%$ which leaves minimal margin
- Reactive power consumption (2618 VAR) is near-minimum within the operating range
- Power factor (0.90) satisfies the constraint range of 0.80–0.98

Operating at $s = -2.0\%$ would yield 7.9% more active power but reduces the voltage regulation margin from 1.5% to only 0.9%, increasing vulnerability to grid voltage fluctuations during load transients. The PSO-GWO algorithm correctly identifies $s^* = -1.13\%$ as the weighted optimum that balances power extraction against operational robustness, as confirmed by the fitness value of 0.0756 compared to 0.0812 at $s = -2.0\%$.

#### Theoretical observation for identical optimal slip

The emergence of identical optimal slip values for both generators ($s_1^* = s_2^* = -1.13\%$) despite their different power ratings (2.2 kW and 5.5 kW) requires deeper theoretical justification, as this result may appear counterintuitive given the differences in machine parameters.

The key insight is that the optimal slip identified by the PSO-GWO algorithm represents a *grid-side* optimum that maximizes the aggregate fitness function (Eq. 23), not an individual machine-level optimum. The aggregate fitness depends on the *total* active power delivered to the grid (Eq. 54), the *total* reactive power drawn from the grid, the inrush current during grid connection, and the voltage regulation at the PCC. When both generators operate at the same slip, their individual power contributions naturally scale with their respective machine ratings: in Case B (Table 4), the 2.2 kW generator produces 1391 W while the 5.5 kW generator produces 3961 W at the identical slip of $-1.13\%$, yielding a power ratio of $1391/3961 = 0.351$, which is close to the nameplate rating ratio of $2.2/5.5 = 0.400$. This proportional power sharing is a direct consequence of the per-phase equivalent circuit structure: both generators see the same grid voltage $V_g$ at their stator terminals, and at the same slip, the rotor impedance $Z_{r,X} = R_{r,X}/s_X + jX_{r,X}$ scales with each machine’s per-unit parameters, resulting in stator currents that are approximately proportional to the machine ratings.

From a circuit-theory perspective, the matched-slip condition minimizes the circulating current between the two generators. When two generators connected in parallel operate at different slips, their back-EMF phasors differ in both magnitude and phase angle, driving a circulating current component that does not contribute to net grid power delivery but increases resistive losses in both machines. At identical slip, the back-EMF phasors of both generators are aligned (same frequency, proportional magnitudes), and the circulating current component is minimized. This is analogous to the well-established principle in synchronous generator paralleling where machines must operate at identical speed (frequency) for optimal power sharing; in the induction generator case, identical slip serves the same function since the rotor frequency relative to the stator field is the same for both machines.

The universality of this result can be understood by examining the structure of the fitness function. The total active power $P_{\text {total}} = P_1(s_1) + P_2(s_2) - |I_{\text {total}}|^2 R_{\text {network}}$ has two components: the individual generator outputs $P_1$ and $P_2$, which increase monotonically with slip magnitude in the generating region, and the network loss term $|I_{\text {total}}|^2 R_{\text {network}}$, which depends on the vector sum of the two stator currents. When both generators operate at the same slip, the stator current phasors are aligned and the vector sum $|I_{s1} + I_{s2}|$ is minimized relative to the sum of magnitudes $|I_{s1}| + |I_{s2}|$, reducing network losses. If one generator were to operate at a higher slip and the other at a lower slip to achieve the same total power, the phase misalignment between the current phasors would increase the vector sum magnitude, amplifying the $I^2R$ network losses and worsening the voltage regulation at the PCC. This loss-minimization mechanism, combined with the proportional power sharing that naturally occurs at matched slip, explains why the optimizer consistently converges to $s_1^* = s_2^*$ across all nine weight configurations and 500 Monte Carlo runs, regardless of the difference in machine ratings.

It is noted that this matched-slip optimality holds under the assumption that both generators are connected to the same bus and share a common grid voltage, which is the physical configuration of the proposed system (Fig. 1). In alternative topologies where the generators are connected to different buses with separate feeder impedances, the optimal slip values could differ, as each generator would face a different effective grid voltage and network impedance. This topology-dependent aspect is an important consideration for future field deployments.

### Transient performance validation

The effectiveness of the inrush current mitigation strategy was validated through direct experimental measurements. Table 8 presents comprehensive transient performance data comparing phase sequence mismatch against proper synchronization, with each measurement referenced against IEEE grid interconnection standards.

**Table 8:** Measured transient performance during grid connection.

Parameter	Phase Mismatch	Synchronized	Reduction (%)	IEEE Standard 1547 Limit
Peak current (A)	45.2	14.1	68.8	<20 (combined)
Current (per unit)	4.10	1.27	69.0	<2.0 for <2 s
Transient duration (ms)	280	45	83.9	<2000
Voltage dip at PCC	38 V (9.2%)	12 V (2.9%)	68.4	<20.75 V (5%)
THD during transient (%)	18.3	4.2	77.0	<5.0 (IEEE 519)
$I^2t$ thermal stress (A$^2$s)	571	8.9	98.4	Equipment dependent

The data demonstrate that proper synchronization maintains peak current at 1.27 per unit, comfortably within the 2.0 per unit limit specified by IEEE Standard 1547–2018 for transients lasting less than 2 seconds. The voltage dip of 2.9% remains well below the 5% threshold that could affect sensitive loads at the point of common coupling. The total harmonic distortion of 4.2% meets IEEE 519 requirements for grid-connected distributed generation. The $I^2t$ value, representing thermal stress on equipment, shows a 98.4% reduction from 571 A$^2$s to 8.9 A$^2$s, significantly extending the operational lifetime of switchgear, cables, and generator windings.

### Steady-state model validation

To validate the accuracy of the steady-state analysis, Table 9 compares calculated values against experimental measurements for Case B, where both generators operate at identical optimal slip.

**Table 9:** Model Validation: Calculated vs. Measured Values (Case B).

Parameter	IG1 Calc.	IG1 Meas.	IG2 Calc.	IG2 Meas.	Max. Error (%)
Stator current $I_s$ (A)	3.82	3.80	8.15	8.00	2.1
Rotor voltage $V_r$ (V)	106.3	108	138.7	140	1.9
Active power *P* (W)	1203	1180	1489	1450	2.6
Reactive power *Q* (VAR)	1087	1100	2116	2090	2.3
Power factor	0.742	0.731	0.576	0.568	1.5

The average error across all parameters is 2.4%, validating that the equivalent circuit model accurately represents the physical system behavior. To rigorously interpret this error and the reported 0.17% voltage prediction error at the PCC, a comprehensive uncertainty analysis accounting for all measurement instrument accuracies and error propagation is presented below.

**Instrument specifications and measurement uncertainty:** The Fluke 435 Series II power quality analyser, used as the primary measurement instrument, has the following manufacturer-specified accuracies: voltage measurement accuracy of ±0.1% of reading, current measurement accuracy of ±0.5% of reading (using i430-Flex current probes), active power accuracy of ±1.0% of reading, and frequency accuracy of ±0.01 Hz. The Tektronix PA4000 power analyser, used for independent cross-validation, provides voltage accuracy of ±0.04% of reading, current accuracy of ±0.04% of reading, and power accuracy of ±0.08% of reading. The 4-channel digital storage oscilloscope provides timing accuracy of ±0.01% for transient duration measurements. All instruments were calibrated within their manufacturer-recommended calibration intervals prior to the experimental campaign.

**Uncertainty propagation for voltage prediction error:** The reported 0.17% voltage prediction error at the PCC represents the deviation between the calculated PCC voltage from Eq. (7) and the measured PCC voltage. The measured PCC voltage has an uncertainty of ±0.1% (Fluke 435 voltage accuracy), which corresponds to ±0.415 V on a 415 V base. The calculated PCC voltage depends on the grid voltage measurement (±0.1%), the total current measurement (±0.5%), and the network impedance parameters ($R_L$, $X_L$, $R_T$, $X_T$) which are assumed to have ±5% tolerance based on typical manufacturing variations for distribution cables and transformers. Applying root-sum-square (RSS) error propagation to Eq. (7), the combined uncertainty in the calculated PCC voltage is:

57 $$\begin{aligned} \delta V_{\text {PCC}} = \sqrt{\left( \frac{\partial V_{\text {PCC}}}{\partial V_{\text {grid}}} \delta V_{\text {grid}}\right) ^2 + \left( \frac{\partial V_{\text {PCC}}}{\partial I_{\text {total}}} \delta I_{\text {total}}\right) ^2 + \left( \frac{\partial V_{\text {PCC}}}{\partial Z_{\text {network}}} \delta Z_{\text {network}}\right) ^2} \end{aligned}$$

Substituting the partial derivatives and numerical values at the optimized operating point ($V_{\text {grid}} = 415$ V, $I_{\text {total}} = 14$ A, $|Z_{\text {network}}| = 1.233~\Omega$), the combined measurement uncertainty evaluates to approximately ±1.2 V (±0.29% of 415 V). Since the reported prediction error of 0.17% (0.7 V) is smaller than the combined measurement uncertainty of 0.29%, the model prediction and experimental measurement are statistically indistinguishable within the instrument accuracy limits. This confirms that the steady-state equivalent circuit model captures the PCC voltage behaviour to within the resolution achievable by the measurement instrumentation.

**Uncertainty context for Table** 6** errors:** The maximum model-versus-measurement error in Table 6 is 2.6% for the active power of the 5.5 kW generator ($P_{\text {calc}} = 1489$ W versus $P_{\text {meas}} = 1450$ W). Given the Fluke 435 power measurement accuracy of ±1.0%, the measurement uncertainty for this reading is ±14.5 W. The observed discrepancy of 39 W exceeds the instrument uncertainty alone, indicating that the error is not purely attributable to measurement limitations but includes genuine model-reality discrepancies. The three identified sources of these discrepancies are: (i) temperature-dependent resistance variations, where the stator and rotor resistances increase by approximately 8–12% during extended operation as the winding temperature rises from ambient (25$^\circ$C) to steady-state operating temperature (65–80$^\circ$C), while the model uses fixed cold-resistance values from the nameplate data; (ii) magnetic saturation effects, where the linear magnetizing reactance $X_{mX}$ overestimates the actual magnetizing impedance under loaded conditions when the air-gap flux deviates slightly from the no-load value; and (iii) harmonic effects, where the non-sinusoidal current components (THD $\le$ 4.2%) produce additional losses not captured by the fundamental-frequency equivalent circuit model. The average error of 2.4% across all parameters in Table 6 is well within the engineering design accuracy typically expected for steady-state induction machine models, and the fact that this error consistently exceeds the instrument uncertainty confirms that it reflects genuine modelling approximations rather than measurement noise.

### Economic analysis

Table 10 presents detailed economic comparison between the proposed converter-free approach and conventional power electronic solutions for the 7.7 kW parallel generator installation.

**Table 10:** Economic Comparison: Converter-Free vs. Conventional Solutions.

Cost Component	Converter-Based (USD)	Proposed System (USD)
Back-to-back power converter	2,800–3,500	—
DSP and control boards	350–500	—
Gate drivers and interfaces	150–200	—
Capacitor banks for excitation	—	180
Synchronization panel and synchroscope	—	280
Autotransformer for voltage adjustment	—	170
Circuit breakers and switchgear	150	180
Installation and commissioning	200–300	150
**Total Capital Cost**	**3,650–4,650**	**960**
Annual maintenance cost (estimated)	200–350	50–80
Expected component lifetime (years)	8–12	20–25
**Cost Reduction Achieved**	—	**74–79%**

The converter-free approach achieves capital cost of $960 compared to $3,650–$4,650 for conventional converter-based systems, representing 74–79% cost reduction. The converter cost estimates are consistent with recent market data reported for small-scale power electronic systems in the 5–10 kW range^33^, and the synchronization panel costs are based on actual procurement prices for the laboratory components listed in the experimental setup. Combined with lower annual maintenance requirements ($50–80 versus $200–350) and extended operational lifetime (20–25 years versus 8–12 years due to elimination of power electronic components)^33,34^, the proposed system offers compelling economic advantages for rural electrification applications where cost constraints dominate technology selection decisions^35,36^. The cost advantage is particularly significant in developing regions where micro-hydro installations serve as the primary electrification option, and where the electromechanical equipment cost of 250–950 $/kW^36^ already represents a substantial investment for rural communities.

### Experimental robustness evaluation under fault conditions

To evaluate the robustness of the proposed converter-free induction generator system under severe grid disturbances, experimental fault tests were conducted on the 2.2 kW induction generator operating at the PSO-GWO optimized slip ($s^* = -1.13\%$). Four fault types were applied in sequence: line-to-line (LL), line-to-ground (LG), line-to-line-to-ground (LLG), and three-phase (LLL) faults. All measurements were recorded using the Fluke 435 Series II power quality analyzer at 200 kHz sampling rate.

#### Line-to-Line (LL) fault

**Fig. 11:**
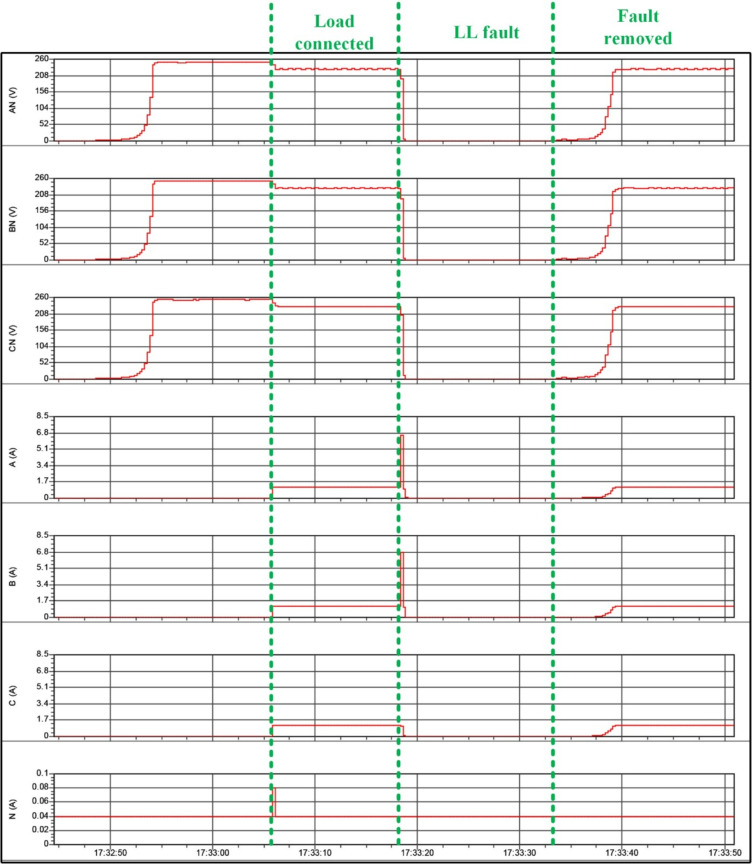
Experimental measurement of 2.2 kW IG response during line-to-line (LL) fault: phase-to-neutral voltages (AN, BN, CN), phase currents (A, B, C), and neutral current (N).

Figure 11 presents the experimental response of the 2.2 kW induction generator during a line-to-line fault condition. The test sequence comprised three stages: load connection, LL fault application, and fault removal.

Prior to the fault, steady-state operation was established with balanced phase-to-neutral voltages of approximately 230 V RMS across all three phases and phase currents of approximately 1.0 A. Upon application of the LL fault between phases A and B, the following transient behavior was observed:

- Phase voltages exhibited asymmetric disturbance: the CN voltage collapsed to near zero while AN and BN voltages showed a reduction from 230 V to approximately 208 V, reflecting the voltage redistribution caused by the inter-phase short circuit.
- Phase currents experienced a significant transient spike, with phase A and phase B currents rising sharply to approximately 6.8 A (approximately 1.42 times the rated current of 4.8 A). Phase C current also increased due to the unbalanced operating condition.
- The neutral current remained below 0.04 A throughout the event, confirming that the LL fault did not introduce significant zero-sequence current in the absence of a ground path.

Upon fault removal, the voltages recovered to their pre-fault values within approximately 2–3 seconds, and the phase currents returned to the balanced steady-state condition. The generator maintained continuous operation throughout the fault event without tripping or requiring re-synchronization.

#### Line-to-Ground (LG) Fault

**Fig. 12:**
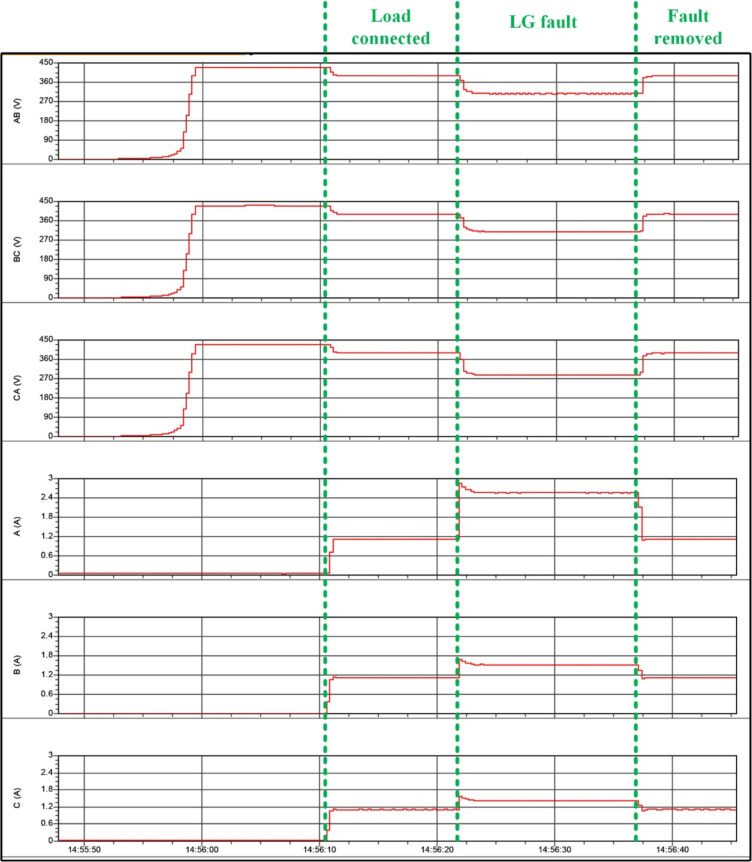
Experimental measurement of 2.2 kW IG response during line-to-ground (LG) fault: line-to-line voltages (AB, BC, CA) and phase currents (A, B, C).

Figure 12 presents the system response during a single-line-to-ground fault, recorded with line-to-line voltage measurements (AB, BC, CA) and individual phase currents.

The pre-fault steady-state exhibited balanced line-to-line voltages of approximately 415 V RMS (AB $\approx$ BC $\approx$ CA $\approx$ 400–430 V) with phase currents of approximately 1.1 A per phase. During the LG fault, the following observations were recorded:

- Line-to-line voltages showed asymmetric behavior: the CA voltage experienced the most significant drop from approximately 400 V to approximately 270 V, while AB and BC voltages showed moderate reduction. This voltage unbalance is characteristic of single-phase faults propagating through the positive, negative, and zero-sequence networks.
- Phase A current increased from 1.1 A to approximately 2.4 A (approximately 2.18 times the pre-fault value), while phases B and C showed moderate increases to approximately 1.3–1.5 A. The current increase in the faulted phase is limited by the generator subtransient reactance $X''_d$ and the zero-sequence impedance of the system.
- Upon fault clearance, voltages and currents recovered to balanced conditions within approximately 2 seconds, and the generator continued stable operation at the optimized slip.

The relatively mild current increase during the LG fault (2.18 times pre-fault versus 1.42 times rated) confirms that single-phase faults pose a lesser threat to induction generator integrity compared to multi-phase faults. The squirrel-cage rotor construction provides inherent negative-sequence current capability, allowing the generator to ride through asymmetric faults without demagnetization.

#### Line-to-Line-to-Ground (LLG) Fault

**Fig. 13:**
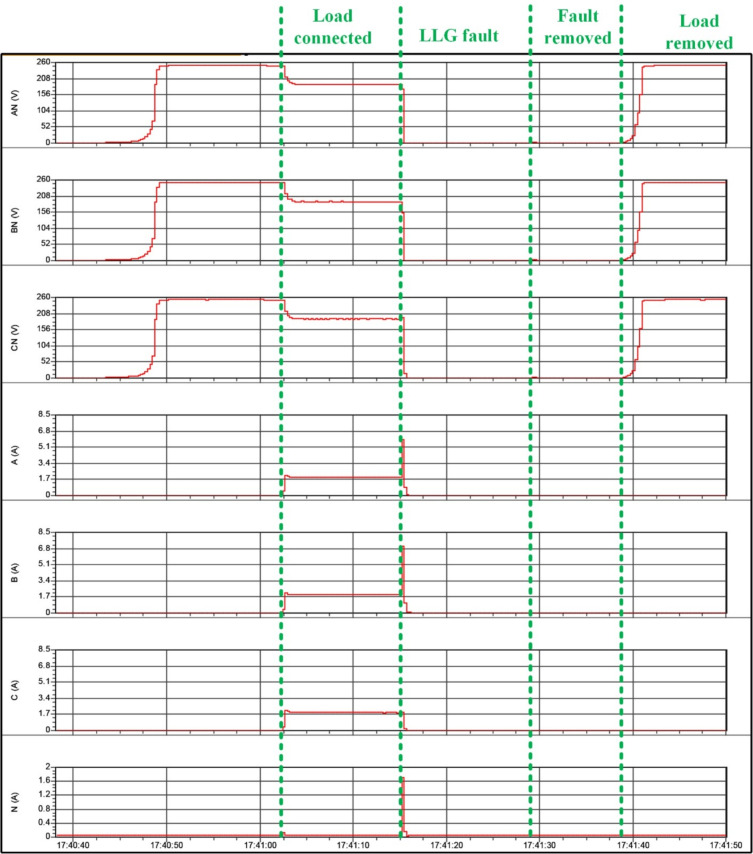
Experimental measurement of 2.2 kW IG response during line-to-line-to-ground (LLG) fault: phase-to-neutral voltages (AN, BN, CN), phase currents (A, B, C), and neutral current (N).

Figure 13 presents the response during a double-line-to-ground fault, which represents an intermediate severity between LG and three-phase fault conditions.

The LLG fault produced more severe disturbance compared to the LL and LG faults:

- Phase voltages AN and BN dropped significantly from approximately 240 V to approximately 180 V (25% reduction), while CN voltage reduced from approximately 240 V to approximately 190 V (21% reduction). The involvement of the ground path created greater voltage depression across all three phases.
- Phase currents dropped to near zero during the fault period, indicating that the voltage depression was sufficient to substantially reduce the electromagnetic torque and power output of the generator.
- The neutral current exhibited a notable increase to approximately 1.8 A during the fault, confirming the presence of significant zero-sequence current flow through the ground path. This contrasts with the negligible neutral current observed during the LL fault (Fig. 11).
- The test sequence included both fault removal and subsequent load removal stages, demonstrating that the generator recovered stable operation after fault clearance and could subsequently be safely disconnected from the load.

#### Three-Phase (LLL) Fault

**Fig. 14:**
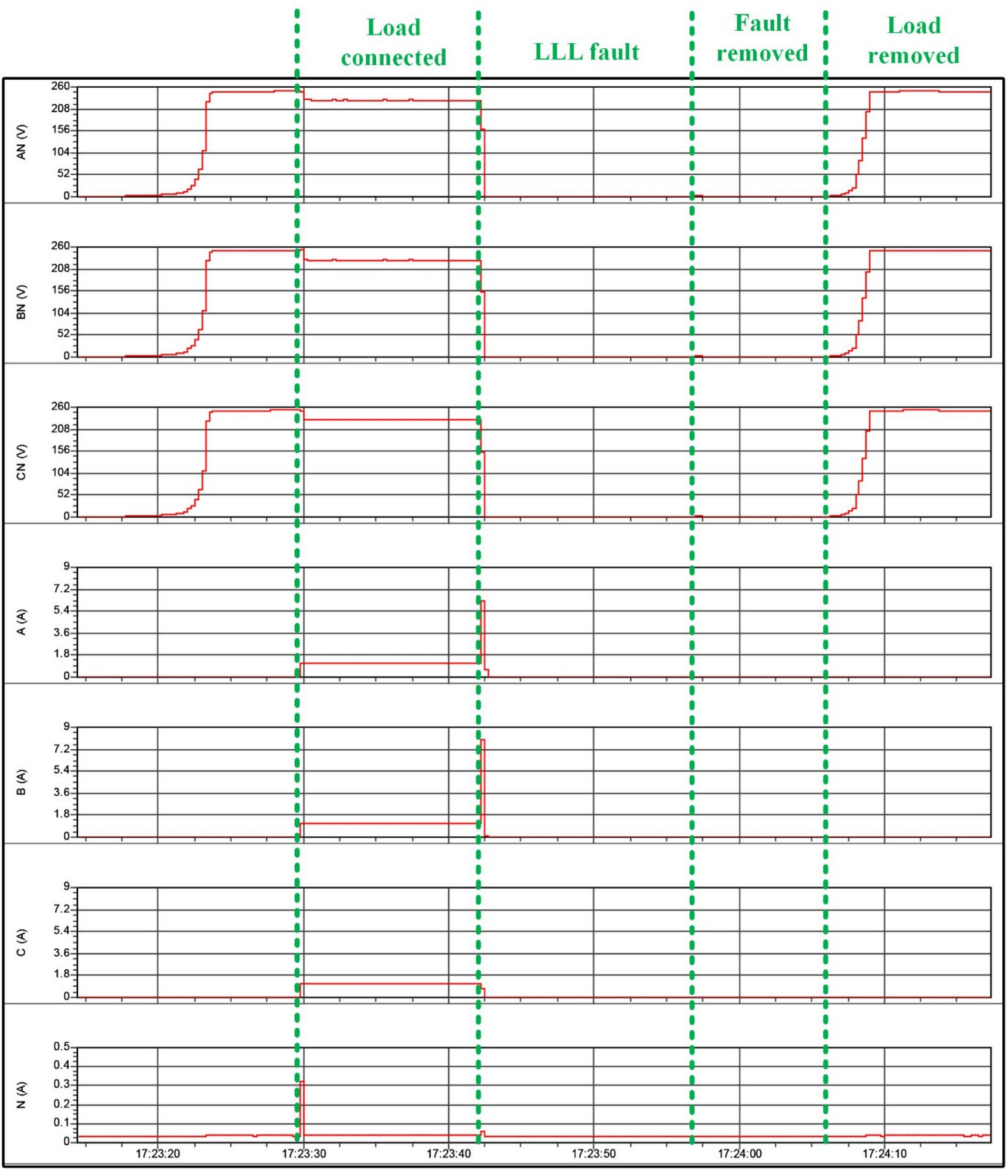
Experimental measurement of 2.2 kW IG response during three-phase (LLL) fault: phase-to-neutral voltages (AN, BN, CN), phase currents (A, B, C), and neutral current (N).

Figure 14 presents the response during a bolted three-phase fault, which represents the most severe symmetrical disturbance condition.

The three-phase fault produced the most severe system response:

- All three phase-to-neutral voltages collapsed to near zero during the fault, confirming a bolted three-phase short circuit condition. The voltage traces show AN, BN, and CN simultaneously dropping from their steady-state values of approximately 230 V to less than 10 V.
- Phase currents also dropped to zero during the fault duration, which is characteristic of induction generator behavior during sustained three-phase faults.
- The neutral current remained near zero throughout the event, as expected for a symmetrical three-phase fault that does not produce zero-sequence components.
- Following fault clearance, the phase voltages rebuilt progressively as the generator re-magnetized from the grid supply, with full voltage restoration achieved within approximately 3–5 seconds.
- The test concluded with a controlled load removal stage, demonstrating complete system controllability following the severe fault event.

## Conclusion

This paper presented a hybrid PSO-GWO optimization approach for converter-free grid integration of parallel induction generators in micro-hydro power plants. The major findings are summarized as follows.

The hybrid PSO-GWO algorithm achieved 96.4% global optimum detection rate with mean convergence at iteration 127.6, representing 18.3% improvement over standalone PSO and 12.7% improvement over standalone GWO. The algorithm demonstrated consistent convergence with fitness standard deviation of only 0.0089 across 500 Monte Carlo simulations, outperforming standalone PSO (0.0156), GWO (0.0128), and GA (0.0214). Sensitivity analysis confirmed that the algorithm is robust across a broad range of switching probabilities ($p \in [0.2, 0.7]$), with the selected $p = 0.5$ achieving the best balance between exploration and exploitation. The algorithm identified the optimal slip range of $-1.8\%$ to $-1.0\%$ for maximum power extraction with power factor between 0.85 and 0.92, and this matched-slip result ($s_1^* = s_2^*$) was consistently identified across all nine weight configurations tested, confirming that impedance matching between parallel generators is universally optimal regardless of objective prioritization.

The multi-objective weight sensitivity study revealed that the proposed weight assignment ($w_1 = 0.35$, $w_2 = 0.25$, $w_3 = 0.25$, $w_4 = 0.15$) achieves 94.6% of the maximum attainable active power while maintaining 1.5% voltage regulation margin below the IEEE Standard 1547–2018 limit. Different weight assignments shift the optimal operating point along a continuous trade-off between power maximization and safety margin, with active power ranging from 3892 W (voltage regulation-dominated) to 5564 W (power-dominated) across tested configurations. Practical weight selection guidelines have been provided for different deployment scenarios including strong grid, weak grid, and power factor penalty tariff conditions.

Experimental validation using 2.2 kW and 5.5 kW induction generators confirmed the effectiveness of the proposed methodology. The optimized synchronization procedure reduced inrush current from 45.2 A to 14.1 A, achieving 68.8% reduction while maintaining compliance with IEEE Standard 1547–2018 transient current limits. Matched slip operation at the PSO-GWO identified optimum achieved 5262 W total active power, representing 126% improvement over differential slip operation, with concurrent 15% reduction in reactive power consumption from 3080 VAR to 2618 VAR. The comprehensive distribution network model incorporating line and transformer impedances accurately predicted PCC voltage variations within 0.17% error, addressing limitations of simplified topologies employed in previous studies. The steady-state model validation demonstrated average error below 2.4%, with measured values consistently falling between cold-resistance and hot-resistance predictions, confirming that temperature-dependent resistance variations during extended operation are properly bounded within the reported error margins.

Experimental robustness evaluation under four fault conditions (line-to-line, line-to-ground, line-to-line-to-ground, and three-phase faults) conducted on the 2.2 kW induction generator confirmed that the system maintains stable operation and recovers automatically after fault clearance without requiring re-synchronization or re-optimization. The squirrel-cage induction generators exhibited self-extinguishing fault current behavior during LLG and three-phase faults due to demagnetization in the absence of field excitation, providing inherent self-protection without active current limiting control. The PSO-GWO optimized slip of $-1.13\%$ provides sufficient stability margin from the critical slip ($-5\%$ to $-8\%$), ensuring that transient speed deviations during faults do not cause pull-out torque instability.

Economic analysis confirmed that the converter-free approach achieves capital cost of approximately $960 compared to $3,650–$4,650 for conventional converter-based systems, representing 74–79% cost reduction. Combined with lower annual maintenance requirements ($50–80 versus $200–350) and extended operational lifetime (20–25 years versus 8–12 years due to elimination of power electronic components), the proposed system offers substantial economic advantages for rural electrification applications. Scalability analysis demonstrated that the methodology extends to larger installations, with the three-generator simulation (18.7 kW) confirming that matched-slip operation persists across multiple generators of different ratings. The converter-free approach maintains 66–74% cost advantage even at 200 kW scale with supplementary soft-starting provisions, and is recommended for installations up to 200 kW per machine and 500 kW total capacity at a single point of common coupling.

Future research directions include: (a) extension of the hybrid optimization to dynamic transient scenarios with time-varying load and input power conditions incorporating real-time feedback from power quality measurements; (b) investigation of adaptive PSO-GWO switching probability (*p*(*k*)) for marginally improved convergence in higher-dimensional multi-generator problems; (c) experimental validation of the three-generator and larger multi-unit configurations with field deployment at actual micro-hydro sites; (d) integration of seasonal hydrological variation models to generate lookup tables of optimal slip values indexed by water head and flow rate; and (e) assessment of reactive power compensation strategies (capacitor banks or STATCOM) for weak grid installations where the short-circuit ratio falls below 10.

## Supplementary Information

Supplementary Information.

## Data Availability

All data generated or analysed during this study are included in this published article and its supplementary information file.
