# Supplementary material for: Hybrid PSO-GWO optimization for converter-free grid integration of parallel induction generators with experimental validation in micro-hydro power plants
Source: Sci Rep. 2026 Apr 20;16:18235. doi: 10.1038/s41598-026-49653-2 (PMC13260900; doi:10.1038/s41598-026-49653-2)
Supplement: Supplementary file 1 — Supplementary Information. [file 41598_2026_49653_MOESM1_ESM.pdf]

Supplementary Material

Hybrid PSO-GWO Optimization for Converter-Free Grid Integration of Parallel Induction Generators with Experimental Validation in Micro-Hydro Power Plants

Mrinal Kanti Rajak[1,*], Ingudam Chitrasen Meitei[1], Rajen Pudur[2]

[1]Department of Electrical Engineering, SVERI's College of Engineering Pandharpur, Maharashtra, India

[2]Department of Electrical Engineering, National Institute of Technology Arunachal Pradesh, India

[*]Corresponding author: mrinal.phd20@nitap.ac.in

1 Extended Machine Parameters and Test Equipment Specifications

1.1 Prime Mover Specifications

Table 1 presents the specifications of the prime movers used to drive the induction generators.

Table 1: Prime Mover (Induction Motor) Specifications

**Parameter**	**PM1 (for IG1)**	**PM2 (for IG2)**
Rated power (kW)	3.7	7.5
Rated voltage (V)	415	415
Rated current (A)	7.5	14.5
Rated speed (RPM)	1440	1460
Efficiency class	IE2	IE2
Power factor at full load	0.84	0.86
Starting current ratio	6.5	6.8

1.2 Detailed Induction Generator Parameters

Table 2 presents comprehensive electrical and mechanical parameters for both induction generators used in the experimental investigation.

Table 2: Comprehensive Induction Generator Parameters

Category	Parameter	IG1 (2.2 kW)	IG2 (5.5 kW)
**Nameplate Data**	Rated power (kW)	2.2	5.5
	Rated voltage (V)	415	415
	Rated current (A)	4.8	11.0
	Rated speed (RPM)	1440	1460
	Rated frequency (Hz)	50	50
	Number of poles	4	4
**Synchronous Data**	Synchronous speed (RPM)	1500	1500
	Rated slip (%)	4.0	2.67
	Slip at optimal operation (%)	−1.13	−1.13
	Speed at optimal operation (RPM)	1517	1517
**Equivalent Circuit**	Stator resistance R_s (Ω)	2.85	1.12
	Rotor resistance R_r (Ω)	2.53	0.98
	Stator leakage reactance X_{ls} (Ω)	3.92	1.85
	Rotor leakage reactance X_{lr} (Ω)	3.92	1.85
	Magnetizing reactance X_m (Ω)	85.6	42.3
	Core loss resistance R_c (Ω)	1250	680
**Mechanical Data**	Moment of inertia J (kg·m^2)	0.012	0.045
	Friction coefficient B (N·m·s)	0.0008	0.0015
	Shaft diameter (mm)	28	38
	Cooling method	TEFC	TEFC
**Excitation Data**	Excitation capacitance per phase (μF)	15	36
	Minimum capacitance for excitation (μF)	12	30
	Capacitor voltage rating (V)	440	440

1.3 Variable Frequency Drive Settings

Table 3 presents the VFD configuration parameters used for speed control.

Table 3: Variable Frequency Drive Configuration

Parameter	VFD1 (3.7 kW)	VFD2 (7.5 kW)
Manufacturer/Model	ABB ACS355	ABB ACS355
Input voltage (V)	380–480	380–480
Output frequency range (Hz)	0–120	0–120
Control mode	V/f with slip compensation	V/f with slip compensation
Carrier frequency (kHz)	8	8
Acceleration time (s)	10	15
Deceleration time (s)	10	15
Speed resolution (%)	0.01	0.01

1.4 Test Equipment Specifications

Table 4 presents detailed specifications of all measurement instruments.

Table 4: Measurement Equipment Specifications

Equipment	Model	Key Specifications
Power Quality Analyzer	Fluke 435 Series II	Sampling: 200 kHz, Accuracy: ±0.5%<br>Voltage range: 1–1000 V RMS<br>Current range: 0.5–6000 A (with clamps)<br>THD measurement: up to 50th harmonic
Digital Storage Oscilloscope	Tektronix TBS2000	Bandwidth: 200 MHz<br>Sampling rate: 2 GS/s<br>Channels: 4<br>Vertical resolution: 8 bits
Current Transducer	LEM LA 55-P	Nominal current: 50 A<br>Accuracy: ±0.65%<br>Bandwidth: DC to 200 kHz
Voltage Transducer	LEM LV 25-P	Nominal voltage: 500 V<br>Accuracy: ±0.8%<br>Bandwidth: DC to 200 kHz
Digital Tachometer	Lutron DT-2236	Range: 2.5–99,999 RPM<br>Accuracy: ±0.05%
Synchroscope	Yokogawa 2170	Frequency range: 45–65 Hz<br>Voltage range: 100–500 V

2 Extended Optimization Data

2.1 Pareto-Optimal Solutions

Table 5 presents the complete set of Pareto-optimal solutions identified by the hybrid PSO-GWO algorithm.

Table 5: Complete Pareto-Optimal Solutions from Hybrid PSO-GWO

**Point**	$\mathbf{s_1}$ (%)	$\mathbf{s_2}$ (%)	$\mathbf{V_{ref}}$ (V)	$\mathbf{P_{total}}$ (W)	$\mathbf{Q_{total}}$ (VAR)	**PF**	**VR** (%)	**Fitness**
1	−2.00	−2.00	435	5679	2745	0.90	4.1	0.0812
2	−1.80	−1.80	428	5534	2698	0.90	3.9	0.0785
3	−1.50	−1.50	424	5423	2665	0.90	3.7	0.0768
4	−1.30	−1.30	422	5342	2638	0.90	3.6	0.0761
**5**	**−1.13**	**−1.13**	**421**	**5262**	**2618**	**0.90**	**3.5**	**0.0756**
6	−1.00	−1.00	420	5108	2589	0.89	3.3	0.0762
7	−0.80	−0.80	418	4812	2634	0.88	3.0	0.0798
8	−0.60	−0.60	416	4456	2712	0.85	2.7	0.0856
9	−0.40	−0.40	414	4012	2798	0.82	2.4	0.0934
10	−0.20	−0.20	412	3524	2876	0.78	2.1	0.1028

2.2 Hybrid PSO-GWO Algorithm Pseudocode

Table 6: Hybrid PSO-GWO Algorithm Pseudocode

**Algorithm: Hybrid PSO-GWO for Parallel IG Optimization**
**Input:** Population size $N = 50$, Max iterations $k_{max} = 200$,
Bounds: $s_{min} = -3\%$, $s_{max} = +1\%$, $V_{min} = 380$ V, $V_{max} = 440$ V
**Output:** Optimal parameters s_1^*, s_2^*, V_{ref}^*
Initialize population $\mathbf{X} = \{x_1, x_2, ..., x_N\}$ randomly within bounds
Initialize velocities $\mathbf{V} = \{v_1, v_2, ..., v_N\}$
Evaluate fitness F_i for all particles using Eq. (fitness function)
Set $p_{best,i} = x_i$ for all i; Identify g_{best}, α, β, δ
**for** $k = 1$ to k_{max} **do**
Update inertia weight: $\omega = 0.9 - 0.5 \times (k/k_{max})$
Update GWO parameter: $a = 2 - 2 \times (k/k_{max})$
**for** $i = 1$ to N **do**
Generate random number $r \in [0, 1]$
**if** $r < 0.5$ **then** // PSO Phase
$v_i = \omega v_i + c_1 r_1 (p_{best,i} - x_i) + c_2 r_2 (g_{best} - x_i)$
$x_i = x_i + v_i$
**else** // GWO Phase
Calculate $\vec{A}_1, \vec{A}_2, \vec{A}_3$ and $\vec{C}_1, \vec{C}_2, \vec{C}_3$
$\vec{D}_\alpha = \lvert\vec{C}_1 \cdot \vec{X}_\alpha - x_i\rvert$
$\vec{D}_\beta = \lvert\vec{C}_2 \cdot \vec{X}_\beta - x_i\rvert$
$\vec{D}_\delta = \lvert\vec{C}_3 \cdot \vec{X}_\delta - x_i\rvert$
$\vec{X}_1 = \vec{X}_\alpha - \vec{A}_1 \cdot \vec{D}_\alpha$
$\vec{X}_2 = \vec{X}_\beta - \vec{A}_2 \cdot \vec{D}_\beta$
$\vec{X}_3 = \vec{X}_\delta - \vec{A}_3 \cdot \vec{D}_\delta$
$x_i = (\vec{X}_1 + \vec{X}_2 + \vec{X}_3)/3$
**end if**
Apply boundary constraints: $x_i = \max(x_{min}, \min(x_{max}, x_i))$
Evaluate fitness F_i
**if** $F_i < F(p_{best,i})$ **then** $p_{best,i} = x_i$ **end if**
**end for**
Update g_{best}; Re-rank and update α, β, δ
**if** $\lvert F^k - F^{k-1}\rvert < \epsilon$ **then break end if**
**end for**
**return** g_{best} as $(s_1^*, s_2^*, V_{ref}^*)$

2.3 Sensitivity Analysis

Table 7 presents sensitivity analysis of optimization performance to algorithm parameters.

Table 7: Sensitivity Analysis of Hybrid PSO-GWO Parameters

**Parameter Variation**	**Mean Iter.**	**Success Rate**	**Best Fitness**	**Std. Dev.**
Population Size Variation (baseline: $N = 50$)				
$N = 20$	158.4	82.6%	0.0789	0.0145
$N = 30$	142.1	89.2%	0.0772	0.0118
$N = 50$ (baseline)	127.6	96.4%	0.0756	0.0089
$N = 75$	118.3	97.8%	0.0754	0.0076
$N = 100$	112.5	98.2%	0.0753	0.0068
Switching Probability Variation (baseline: $P_{switch} = 0.5$)				
$P_{switch} = 0.2$ (more GWO)	134.8	93.2%	0.0768	0.0102
$P_{switch} = 0.3$	131.2	94.8%	0.0762	0.0095
$P_{switch} = 0.5$ (baseline)	127.6	96.4%	0.0756	0.0089
$P_{switch} = 0.7$	132.4	95.1%	0.0759	0.0092
$P_{switch} = 0.8$ (more PSO)	138.6	92.8%	0.0771	0.0108
Cognitive/Social Coefficient Variation (baseline: $c_1 = c_2 = 2.0$)				
$c_1 = c_2 = 1.5$	145.2	91.4%	0.0778	0.0124
$c_1 = c_2 = 2.0$ (baseline)	127.6	96.4%	0.0756	0.0089
$c_1 = c_2 = 2.5$	135.8	93.6%	0.0765	0.0098
$c_1 = 2.5, c_2 = 1.5$	142.3	92.1%	0.0774	0.0112

2.4 Convergence Data at Selected Iterations

Table 8 presents detailed convergence data at selected iterations for all algorithms.

Table 8: Detailed Convergence Data at Selected Iterations

**Iter.**	**PSO**		**GWO**		**GA**		**Hybrid**	
	Fitness	Std.	Fitness	Std.	Fitness	Std.	Fitness	Std.
1	0.4500	0.0850	0.4500	0.0820	0.4500	0.0890	0.4500	0.0780
10	0.3850	0.0720	0.3620	0.0680	0.4100	0.0810	0.3200	0.0580
25	0.2890	0.0580	0.2540	0.0510	0.3330	0.0690	0.1935	0.0420
50	0.1750	0.0420	0.1440	0.0360	0.2280	0.0540	0.0980	0.0280
75	0.1220	0.0310	0.0960	0.0240	0.1690	0.0420	0.0768	0.0165
100	0.1010	0.0245	0.0840	0.0185	0.1380	0.0350	0.0756	0.0092
125	0.0920	0.0198	0.0812	0.0138	0.1190	0.0295	0.0756	0.0089
150	0.0860	0.0168	0.0812	0.0128	0.1110	0.0258	0.0756	0.0089
175	0.0847	0.0158	0.0812	0.0128	0.1000	0.0228	0.0756	0.0089
200	0.0847	0.0156	0.0812	0.0128	0.0923	0.0214	0.0756	0.0089

3 Extended Experimental Data

3.1 Case A: Detailed Event Log

Table 9 presents the complete event log for Case A experimental procedure.

Table 9: Case A: Complete Event Log with Measured Parameters

Event	Description	V (V)	I (A)	P (W)	Q (VAR)	f (Hz)	PF
0	System initialization	0	0.0	0	0	–	–
15	IG1 voltage build-up start	285	0.8	–	–	48.2	–
20	IG1 voltage established	412	1.2	–	–	49.5	–
30	IG2 voltage build-up start	290	1.5	–	–	47.8	–
38	IG2 voltage established	418	2.1	–	–	49.8	–
60	Individual loads connected	408	4.2	1850	1420	49.6	0.79
62	Parallel synchronization	410	5.8	2180	2650	49.8	0.63
80	Parallel operation stable	412	6.5	2250	2780	49.9	0.63
120	Grid connection	415	9.0	2328	3080	50.0	0.60
140	Load & capacitor disconnection	415	8.8	2310	2950	50.0	0.62
160	Steady-state grid-connected	415	9.0	2328	3080	50.0	0.60

3.2 Case B: Detailed Event Log

Table 10 presents the complete event log for Case B (optimized condition).

Table 10: Case B: Complete Event Log with Measured Parameters (Optimized Condition)

Event	Description	V (V)	I (A)	P (W)	Q (VAR)	f (Hz)	PF
0	System initialization	0	0	0	0	–	–
4	IG1 voltage build-up start	280	0.7	–	–	47.5	–
10	IG1 voltage established	415	1.1	–	–	49.8	–
15	IG2 voltage build-up start	295	1.4	–	–	48.2	–
25	IG2 voltage established	420	2.0	–	–	50.1	–
34	Individual loads connected	410	5.5	2450	1850	49.7	0.80
62	Parallel synchronization	408	8.2	3680	2450	49.9	0.83
86	Grid connection	390	12.5	4820	2580	50.0	0.88
100	Autotransformer adjustment	405	13.2	5050	2610	50.0	0.89
119	Load & capacitor disconnection	412	13.8	5180	2600	50.0	0.89
127	Voltage fully compensated	415	14.0	5262	2618	50.0	0.90
150	Steady-state optimized	415	14.0	5262	2618	50.0	0.90

3.3 Case C: Detailed Speed Variation Data

Table 11 presents comprehensive data for Case C at each speed setting.

Table 11: Case C: Detailed Performance at Each Speed Setting

Parameter	1480 RPM ($s = +1.33\%$)	1499 RPM ($s = -0.07\%$)	1517 RPM ($s = -1.13\%$)	1530 RPM ($s = -2.00\%$)
5.5 kW Induction Generator				
Stator voltage (V)	386	392	421	424
Stator current (A)	1.44	4.10	10.80	11.69
Active power (W)	1902	2707	3961	4711
Reactive power (VAR)	1725	1680	1561	1576
Power factor	0.81	0.79	0.93	0.95
Efficiency (%)	78.2	82.5	89.4	91.2
2.2 kW Induction Generator (constant speed 1517 RPM)				
Stator voltage (V)	386	392	421	424
Stator current (A)	3.2	3.5	4.2	4.1
Active power (W)	966	1012	1391	1067
Reactive power (VAR)	1047	1118	1035	1148
Power factor	0.68	0.67	0.80	0.68
Grid Total				
Total active power (W)	2820	3662	5262	5679
Total reactive power (VAR)	2787	2812	2618	2745
Combined power factor	0.71	0.79	0.90	0.90
Grid current (A)	5.2	8.4	14.0	15.0
PCC voltage (V)	412	414	415	415
Voltage regulation (%)	1.8	2.2	3.5	4.1

3.4 PCC Voltage Variation Analysis

Table 12 presents PCC voltage variation with power output.

Table 12: PCC Voltage Variation with Power Output

P_{total} (W)	Q_{total} (VAR)	I_{total} (A)	Calculated V_{PCC} (V)	Measured V_{PCC} (V)	Error (%)	VR_{PCC} (%)
1378	2856	4.4	419.2	418.8	0.10	0.8
2328	3080	5.4	417.5	417.1	0.10	1.2
2820	2787	5.5	415.8	415.2	0.14	1.5
3662	2812	6.4	412.4	411.8	0.15	2.2
5262	2618	8.1	406.8	406.2	0.15	3.5
5679	2745	8.7	403.2	402.5	0.17	4.1

Appendix A: Nomenclature

Roman Symbols

$\mathbf{A}$	Coefficient matrix of the steady-state equivalent circuit equations (8×8 complex)
a	Phase rotation operator ($a = 1\angle 120$)
$\mathbf{B}$	Right-hand side vector of the matrix equation $\mathbf{AX} = \mathbf{B}$
c_1, c_2	PSO cognitive and social acceleration coefficients
$\vec{C}$	GWO coefficient vector ($\vec{C} = 2 \cdot \vec{r}_2$)
d	Dimension of the search space ($d = 3$)
E	Induced electromotive force (V)
F	Per-unit frequency
F_{total}	Scalar aggregate fitness function
F^k_{best}	Best fitness value at iteration k
f_1, f_2, f_3, f_4	Objective functions: active power, reactive power, inrush current, voltage regulation
$\hat{f}_k$	Min-max normalized objective function value
$g_1, \ldots, g_5$	Inequality constraint violation functions
$\mathbf{g}_{\text{best}}$	Global best position vector in PSO
I_{sX}	Stator current of generator X (A)
I_{rX}	Rotor current of generator X (A)
I_{mX}	Magnetizing current of generator X (A)
I_{total}	Total current injected into the grid (A)
I_{inrush}	Inrush current magnitude during grid connection (A)
$I_{\text{rated},i}$	Rated stator current of generator i (A)
k	Iteration index
$k_{\max}$	Maximum number of iterations ($k_{\max} = 200$)
l	Feeder length (km)
N	Population size ($N = 50$)
N_s	Synchronous speed (RPM)
$N_{r,X}$	Rotor speed of generator X (RPM)
P_X	Active power output of generator X (W)
P_{total}	Total active power delivered to the grid (W)
P_{grid}	Active power at the grid after network losses (W)
PF_X	Power factor of generator X
$\mathbf{p}_{\text{best},i}$	Personal best position of particle i in PSO
Q_X	Reactive power of generator X (VAR)
Q_{total}	Total reactive power consumed from the grid (VAR)
r_1, r_2	Random numbers uniformly distributed in $[0, 1]$
r_L	Distribution line resistance per unit length (Ω/km)
R_L	Total distribution line resistance (Ω)
R_T	Transformer resistance referred to LV side (Ω)

R_{sX}	Stator resistance of generator X (Ω)
R_{rX}	Rotor resistance of generator X (Ω)
R_{network}	Total network resistance (Ω)
s_i	Slip of generator i (per unit or %)
s_1^*, s_2^*	Optimal slip values identified by the hybrid PSO-GWO algorithm
$s_{\min}, s_{\max}$	Lower and upper bounds of the slip search space
S_X	Complex power of generator X (VA)
S_{rated}	Rated apparent power of the transformer (VA)
T_{eval}	Computational cost per fitness evaluation
v	Per-unit rotor speed
v_i^k	Velocity of particle i at iteration k in PSO
V_g	Per-phase grid voltage (V)
V_{grid}	Line-to-line grid voltage (V)
V_{PCC}	Voltage at the point of common coupling (V)
V_{ref}	Voltage reference setpoint (V)
V_{ref}^*	Optimal voltage reference (V)
V_{diff}	Voltage difference between generators at connection instant (V)
V_{rated}	Rated voltage of the transformer (V)
V_{rX}	Rotor voltage of generator X (V)
VR_{PCC}	Voltage regulation at the point of common coupling (%)
VR_{target}	Target voltage regulation (%)
w_1, w_2, w_3, w_4	Weighting factors for the multi-objective fitness function
$\mathbf{x}$	Decision variable vector $[s_1, s_2, V_{\text{ref}}]^T$
x_i^k	Position of particle i at iteration k
$\vec{X}_\alpha, \vec{X}_\beta, \vec{X}_\delta$	Positions of alpha, beta, and delta wolves in GWO
x_L	Distribution line reactance per unit length (Ω/km)
X_L	Total distribution line reactance (Ω)
X_T	Transformer reactance referred to LV side (Ω)
X_{sX}	Stator reactance of generator X (Ω)
X_{rX}	Rotor reactance of generator X (Ω)
X_{mX}	Magnetizing reactance of generator X (Ω)
$\mathbf{X}$	Unknown state vector $[I_{s1}, I_{r1}, I_{m1}, V_{r1}, I_{s2}, I_{r2}, I_{m2}, V_{r2}]^T$
Z_{base}	Base impedance of the transformer (Ω)
Z_{line}	Distribution line impedance (Ω)
Z_T	Transformer impedance (Ω)
Z_{network}	Total network impedance (Ω)
$Z_{r,X}$	Rotor impedance of generator X (Ω)

Z_s	Stator impedance (Ω)
$Z_{\text{total},X}$	Total impedance of generator X (Ω)
Z_{IG1}, Z_{IG2}	Generator impedances during grid connection (Ω)

Greek Symbols

$\vec{a}$	GWO control parameter, linearly decreasing from 2 to 0
$\vec{A}$	GWO coefficient vector ($\vec{A} = 2\vec{a} \cdot \vec{r}_1 - \vec{a}$)
$\vec{D}_\alpha$	Distance between a wolf and the alpha wolf in GWO
λ	Penalty coefficient for constraint violations ($\lambda = 1000$)
λ_s	Stator flux linkage (Wb)
ω	PSO inertia weight
$\omega_{\max}, \omega_{\min}$	Maximum and minimum inertia weight (0.9 and 0.4)
ω_e	Synchronous electrical angular velocity (rad/s)
θ	Transformation angle (rad)
Φ	Magnetic flux (Wb)

Abbreviations

AGWOPSO	Adaptive Grey Wolf Optimizer–Particle Swarm Optimization
DSO	Digital Storage Oscilloscope
DSP	Digital Signal Processor
FFA	Fuzzified Firefly Algorithm
GA	Genetic Algorithm
GWO	Grey Wolf Optimizer
HCHOPSO	Hybrid Cheetah–Particle Swarm Optimization
HFPSO	Hybrid Firefly–Particle Swarm Optimization
IG	Induction Generator
LG	Line-to-Ground (fault type)
LL	Line-to-Line (fault type)
LLG	Line-to-Line-to-Ground (fault type)
LLL	Three-Phase (fault type)
PCC	Point of Common Coupling
PSO	Particle Swarm Optimization
PSO-AHA	Hybrid PSO–Artificial Hummingbird Algorithm
PSO-GWO	Hybrid Particle Swarm Optimization–Grey Wolf Optimizer
RMS	Root Mean Square
RPM	Revolutions Per Minute
SEIG	Self-Excited Induction Generator
STATCOM	Static Synchronous Compensator
THD	Total Harmonic Distortion
VFD	Variable Frequency Drive

Subscripts and Superscripts

$X \in \{1, 2\}$	Generator index (1 = 2.2 kW IG, 2 = 5.5 kW IG)
i	Particle index in PSO/GWO population
j	Decision variable index or constraint index
k	Iteration index
$*$	Optimal value (e.g., s_1^*, V_{ref}^*)
α, β, δ	Alpha, beta, and delta wolves in GWO hierarchy
